# Adoptive Cellular Therapy with Autologous Tumor-Infiltrating Lymphocytes and T-cell Receptor-Engineered T Cells Targeting Common p53 Neoantigens in Human Solid Tumors

**DOI:** 10.1158/2326-6066.CIR-22-0040

**Published:** 2022-08-03

**Authors:** Sanghyun P. Kim, Nolan R. Vale, Nikolaos Zacharakis, Sri Krishna, Zhiya Yu, Billel Gasmi, Jared J. Gartner, Sivasish Sindiri, Parisa Malekzadeh, Drew C. Deniger, Frank J. Lowery, Maria R. Parkhurst, Lien T. Ngo, Satyajit Ray, Yong F. Li, Victoria Hill, Maria Florentin, Robert V. Masi, Biman C. Paria, Noam Levin, Alakesh Bera, Elizabeth A. Hedges, Agnes Choi, Praveen D. Chatani, Anup Y. Parikh, Shoshana Levi, Samantha Seitter, Yong-Chen Lu, Zhili Zheng, Todd D. Prickett, Li Jia, Jonathan M. Hernandez, Chuong D. Hoang, Paul F. Robbins, Stephanie L. Goff, Richard M. Sherry, James C. Yang, Steven A. Rosenberg

**Affiliations:** 1 Surgery Branch, Center for Cancer Research, National Cancer Institute, National Institutes of Health, Bethesda, Maryland.; 2 Laboratory of Pathology, National Cancer Institute, Bethesda, Maryland.; 3 National Institutes of Health Library, Office of Director, National Institutes of Health, Bethesda, Maryland; 4 Surgical Oncology Program, National Cancer Institute, National Institutes of Health, Bethesda, Maryland.; 5 Thoracic Surgery Branch, National Cancer Institute, NIH, CCR and The Clinical Center, Bethesda, Maryland.

## Abstract

Adoptive cellular therapy (ACT) targeting neoantigens can achieve durable clinical responses in patients with cancer. Most neoantigens arise from patient-specific mutations, requiring highly individualized treatments. To broaden the applicability of ACT targeting neoantigens, we focused on *TP53* mutations commonly shared across different cancer types. We performed whole-exome sequencing on 163 patients with metastatic solid cancers, identified 78 who had *TP53* missense mutations, and through immunologic screening, identified 21 unique T-cell reactivities. Here, we report a library of 39 T-cell receptors (TCR) targeting *TP53* mutations shared among 7.3% of patients with solid tumors. These TCRs recognized tumor cells in a *TP53* mutation- and human leucocyte antigen (HLA)-specific manner *in vitro* and *in vivo*. Twelve patients with chemorefractory epithelial cancers were treated with *ex vivo*-expanded autologous tumor-infiltrating lymphocytes (TIL) that were naturally reactive against *TP53* mutations. However, limited clinical responses (2 partial responses among 12 patients) were seen. These infusions contained low frequencies of mutant p53–reactive TILs that had exhausted phenotypes and showed poor persistence. We also treated one patient who had chemorefractory breast cancer with ACT comprising autologous peripheral blood lymphocytes transduced with an allogeneic HLA-A*02–restricted TCR specific for p53^R175H^. The infused cells exhibited an improved immunophenotype and prolonged persistence compared with TIL ACT and the patient experienced an objective tumor regression (−55%) that lasted 6 months. Collectively, these proof-of-concept data suggest that the library of TCRs targeting shared p53 neoantigens should be further evaluated for the treatment of patients with advanced human cancers.

## Introduction

Neoantigens arising from somatic tumor mutations can be successfully targeted by adoptive cellular therapy (ACT) using autologous T cells, leading to long-lasting clinical responses in patients with metastatic cancers (1–4). Thus far, however, the majority of neoantigens identified across different cancer types are encoded by private mutations that are present in tumors from a single patient, which necessitates an individualized treatment for each patient that is time and cost-prohibitive (5–7).

To overcome this issue, we focused on *TP53* (which encodes p53), a key tumor suppressor gene that represents the most commonly mutated gene across different cancer types (8), including the solid epithelial cancers that account for more than 90% of cancer deaths in the United States (9). No current therapeutic agents specifically target *TP53*-mutated cancers. We and others have previously reported that some “hotspot” *TP53* mutations are immunogenic as they can be recognized by human T cells (10–13) or by a synthetic antibody (14). More specifically, Malekzadeh and colleagues reported immunogenicity of “hotspot” *TP53* mutations, such as R175H, Y220C, and R248W (10). In this study, we report immunogenicity of additional *TP53* mutations, including both “hotspot” and “non-hotspot” mutations. We established a library of T-cell receptors (TCR) specific for the mutant p53 proteins and characterized the antitumor activity of some of these TCRs using various *in vitro* and *in vivo* models. Lo and colleagues reported a single case study of treating a patient with metastatic colon cancer with autologous TIL that contained low levels of mutant p53 reactivities (12). In this study, to expand upon our experience of targeting p53-mutated cancers, we treated 11 additional patients with autologous TIL and 1 patient with peripheral blood lymphocytes (PBL) genetically engineered with a TCR targeting the p53^R175H^ neoantigen.

## Materials and Methods

### Overview of the study

In this study, patients with advanced solid cancers were recruited to evaluate the presence of T cells reactive against p53 neoantigens within TILs for potential ACT. Somatic mutations in individual tumors were identified by whole-exome sequencing (WES)/whole transcriptome analysis [RNA sequencing (RNA-seq)]. TILs from the tumors were expanded *ex vivo* and screened for neoantigen reactivity: TILs were cocultured with autologous antigen-presenting cells (APC) that were loaded with mutant peptides or transfected with RNA encoding patient-specific mutations. When reactive TILs were identified, they were further expanded for autologous TIL treatment. 12 patients received autologous TIL ACT that contained mutant p53-reactive T cells. 1 patient with advanced breast cancer whose TIL did not contain mutant p53–reactive T cells received autologous PBL engineered with an allogeneic TCR targeting the p53^R175H^ neoantigen (See also Supplementary Fig. S1).

### Subjects and clinical protocols

Written, informed consent was obtained from all study participants, and all studies were conducted in accordance with The Declaration of Helsinki, The Belmont Report, and the U.S. Common Rule. This study was approved by the Investigational Review Board at the NCI in accordance with an assurance filed with and approved by the U.S. Department of Health and Human Services and was registered at https://clinicaltrials.gov under NCT00068003, NCT01174121, and NCT03412877.

We enrolled 163 patients with chemorefractory metastatic epithelial cancers in the tissue procurement protocol NCT00068003. Metastases and leukaphereses were collected from each patient at the time of recruitment. TILs were expanded *ex vivo* for 2 to 4 weeks, frozen, and kept in liquid nitrogen until use. Leukaphereses were instantly frozen and kept in liquid nitrogen until use. Healthy donors were recruited under the tissue procurement protocol NCT00068003 and underwent leukaphereses.

Adults ages 18 to 70 with upper or lower gastrointestinal, hepatobiliary, genitourinary, breast, or ovarian/endometrial cancer (both NCT01174121 and NCT03412877), or glioblastoma (NCT01174121) or endocrine tumor, neuroendocrine tumor (NCT03412877) refractory to standard chemotherapy were recruited. Patients were treated with either ACT of autologous TILs (NCT01174121) or TCR-engineered autologous PBLs (NCT03412877). Among the 163 patients, 97 patients whose tumor harbored mutations in *TP53* were screened for reactivity against individual patient-specific neoantigens, including p53 neoantigens. Finally, 12 patients were selected for autologous TIL ACT based on the screening results, the availability of TILs, and the patients’ eligibility for the clinical trials at the time of treatment (see Supplementary Fig. S1).

### WES, RNA-seq, and data analysis

Identification of somatic tumor mutations, including *TP53* mutations, by WES and RNA-seq analysis for 41 patients has been previously described (refs. 5, 10, 12, 15, 16; dbGap accession number phs001003.v2.p1). Here, we report the same analysis for an additional 37 patients. In brief, genomic DNA (gDNA) and total RNA were purified from fresh tumors and matched normal apheresis samples using the AllPrep DNA/RNA kit (catalog no. 80204, QIAGEN) according to the manufacturer’s instructions. Whole-exome library construction and exon capture of approximately 20,000 coding genes was performed using SureSelect XT HS Target Enrichment System (catalog no. G9706N, Agilent Technologies) for paired-end libraries and Human All Exon V7 RNA bait (catalog no. 5190–8863, Agilent Technologies) for RNA-seq libraries. The WES library was prepped using gDNA (200 ng) isolated from the fresh tumor tissue following the manufacturer’s protocol. Paired-end sequencing was done on NextSeq 550 desktop sequencers (Illumina) with an Illumina High-output flow cell kit (300 cycles; catalog no. 20024908) using v2.5 of reagent/flow cell kit. RNA-seq libraries were prepared using 100 to 200 ng of total RNA (DNaseI-treated) with the TruSeq RNA Exome library prep kit (catalog nos. 20020189, 20020490, and 20020492, Illumina) following the manufacturer’s protocol. RNA-seq libraries were paired-end sequenced on NextSeq 550 desktop sequencers (Illumina) as described above to generate more than 30 million paired-end reads on average. Alignment to human genome (hg19) was performed by novoalign MPI (Novocraft). Duplicates were marked by Picard’s MarkDuplicates. Insertion and deletion realignment and base recalibration were performed following the GATK best practices workflow (https://www.broadinstitute.org/gatk/). Varscan2, (http://varscan.sourceforge.net), SomaticSniper (http://gmt.genome.wustl.edu/packages/somatic-sniper/), Strelka (https://sites.google.com/site/strelkasomaticvariantcaller/), and Mutect (https://www.broadinstitute.org/gatk/) were used to identify variants. Next, VCF files were merged using GATK CombineVariants tools and annotated using Annovar (http://annovar.openbioinformatics.org). Somatic copy-number alterations were determined using sequenza R package with a mutation frequency threshold adjusted to 0.08 from the default of 0.1 to account for intratumor heterogeneity and normal cell contamination (17).

### Human leucocyte antigen typing and haplotype-specific copy number analysis of human leucocyte antigen loci

WES data of tumor and germline (peripheral blood) samples for each patient were mapped to human reference genome (hg19) using Novoalign (Novocraft). Cellularity and purity of these samples were estimated using Sequenza (17). Human leucocyte antigen (HLA) typing of patients’ germline data was computationally determined by taking consensus of predictions from two HLA-typing algorithms, HLA_PRG_LA (18), and PHLAT (19). With patient’s HLA-typing, cellularity and ploidy estimates, and tumor and germline BAM files, we checked for LOH in the HLA class I alleles using an adjusted version of the original LOHHLA tool (20). This custom version of the LOHHLA tool is deposited in Bitbucket (https://bitbucket.org/SENTISCI/lohhla/src/master/).

### Tumor cell lines

COS7, CEM/C1, HCC1395, SK-MEL5, SAOS2, U-698-M, HCC2935, TYK-nu, KLE, SAOS2-R175H, and SAOS2-Y220C tumor cell lines were previously described (10, 12, 15). Pancreatic adenocarcinoma BXPC3 cell line (catalog no. CRL-1687, RRID:CVCL_0186) and breast adenocarcinoma MCF-7 cell line (catalog no. HTB-22, RRID:CVCL_0031) were purchased from ATCC. All of the above cell lines were purchased between year 2016 and 2018, and passages between 5 and 20 were used. Breast ductal carcinoma JIMT-1 cell line was purchased from DSMZ (catalog no. ACC-589, RRID: CVCL_2077) in 2021 and passages between 5 and 8 were used. *Mycoplasma* testing and cell authenticity were not independently performed as we relied on the commercial vendor’s testing. 4259, 4350, 4324, 4316, and 4247 patient-derived xenograft (PDX) cell lines were initially implanted into NOD-*scid* IL2Rg^null^ (NSG) mice and later established into cell lines that were propagated in culture as described previously (10). Tumor cell lines were cultured in RPMI1640 media (catalog no. 11875093) containing 10% FBS (catalog no. SH30071.03HI), 1% sodium pyruvate (catalog no. 11360070), 1% Glutamax (catalog no. 35050061), 1% minimum essential medium (MEM) nonessential amino acids (catalog no. 11140050), 55 μmol/L 2-mercaptoethanol (catalog no. 21985023), and 1% Pen/Strep (catalog no. 15140122; all of the above from Gibco except for FBS, Cytiva).

### Primary cell cultures

Autologous TILs, immature dendritic cells (imDC), and B cells were obtained as described previously (4, 15). TILs were maintained in 50/50 media [RPMI1640 media containing 10% human serum; (catalog no. HS1021HI, Valley Biomedical), 1% Glutamax, 12.5 mmol/L HEPES (catalog no. 15630080, Gibco), 1% Pen/Strep, and 5 μg/mL gentamicin (catalog no. 120–099–661, Quality Biological) mixed with AIM-V (catalog no. 0870112BK, Gibco) at 1:1 ratio] supplemented with 3,000 IU/mL IL2 (Aldesleukin, National drug code: 76310–0022–01, Clinigen). imDCs were generated and maintained in RPMI1640 media containing 5% human serum, 1% Glutamax, 800 IU/mL GMCSF (Leukine, National drug code: 71837–5843–5, Partner Therapeutics), and 200 IU/mL IL4 (catalog no. 200–04, Peprotech). B cells were generated and maintained in Iscove’s modified Dulbecco’s medium (IMDM; catalog no. 31980030, Gibco) supplemented with 10% human serum, 1% Glutamax, and 200 IU/mL IL4.

### Coculture of T cells with APCs or tumor cells

Screening of TILs for reactivity against *TP53* mutations was performed as described previously (15). Briefly, imDCs or B cells were electroporated with 5 to 10 μg of *in vitro*–transcribed tandem minigene (TMG) RNA that encoded 10 to 15 consecutive mutated 25 mers with the mutation in the middle (1 × 10^5^ cells/well) and rested overnight or pulsed with mutant peptides (5 × 10^4^ imDCs or 1 × 10^5^ B cells/well) for 2 to 4 hours. Differential primary cell counting was done based on cell size using an AOPI dye (catalog no. CS2–0106–25ML, Nexcelom Bioscience) and Auto T4 or Cellaca MX cell counter (Nexcelom Bioscience). 5 to 10 × 10^4^ target cells (imDCs or B cells) were washed twice and resuspended in 50/50 media and cocultured with 2–3 × 10^4^ TILs in IFNγ ELISpot plates [96-well plates with a polyvinylidene difluoride (PVDF) membrane; catalog no. MAIPSWU10, EMD Millipore]. Phorbol 12-myristate 13-acetate (PMA; 81 nmol/L) and ionomycin (1.34 μmol/L; catalog no. 00–4970–93, Thermo Fisher Scientific) were included as a positive control. Cocultured cells were stained and analyzed by flow cytometry as described below (see Antibodies, flow cytometry, and FACS), and IFNγ ELISpot plates were processed using the Human IFNγ ELISpot BASIC kit [horseradish peroxidase (HRP); catalog no. 3420–2H, Mabtech) according to the manufacturer’s instructions.

The ability of TCRs specific for p53 neoantigens (Table 1) to recognize tumor cells was evaluated by coculturing T cells with tumor cells that expressed cognate p53 neoantigens or HLAs with PBLs transduced with TCRs. Tumor cells were detached using TrypLe Express (catalog no. 12605010, Gibco), counted, and cocultured at a 1:1 to 2:1 (target:effector) ratio with T cells (2 to 10 × 10^4^ cells/well) overnight in flat-bottom 96-well plates. Following coculture, supernatant was analyzed to assess IFNγ secretion by ELISA (catalog no. EHIFNG, Thermo Fisher Scientific) and the cocultured cells were analyzed by flow cytometry as described below.

When a TCR reactive to p53 neoantigens was identified, the TCR was cloned into a retroviral vector. Retrovirus was generated and healthy donor–derived PBLs were transduced with the retroviral vector (see TCR cloning and retroviral T-cell transduction). The avidity and the specificity for the p53 neoantigen of the TCR was tested by coculturing the healthy donor PBLs expressing TCRs with autologous APCs pulsed with serially diluted mutated or wildtype (WT) peptides (custom peptide synthesis by Genscript), and then analyzing by flow cytometry and the IFNγ ELISpot assay, as described above.

Determination of HLA for a TCR reactive to a p53 neoantigen was previously described (11). Briefly, COS7 cells lacking HLA expression were individually transfected with plasmids expressing HLAs identified from a patient using lipofectamine 2000 (catalog no. 11668027, Thermo Fisher Scientific) per the manufacturer’s instructions. 2 days later, the transfected COS7 cells were incubated with cognate peptides (1 μg/mL) for 2 to 4 hours, cocultured with T cells expressing the TCR, and analyzed by flow cytometry and the IFNγ ELISpot assay as described above.

The minimal epitope for HLA class I–restricted TCRs was determined as follows. Candidate minimal epitopes were synthesized (Genscript) based on the prediction algorithm NetMHCpan - 4.0 (21) with %rank < 2 as the cutoff. PBLs expressing the TCR of interest were cocultured with autologous APCs pulsed with individual candidate minimal epitopes and were analyzed by flow cytometry and the IFNγ ELISpot assay as described above.

### Generation of clinical TIL infusion products

Generation of clinical TIL infusion products for ACT has been described previously (2,4). Briefly, 24 TIL fragment subcultures were *ex vivo*–expanded for 2 to 4 weeks with IL2 (6,000 IU/mL). Following neoantigen screening as described in the “Coculture of T cells with APCs or tumor cells” section, TIL subcultures with highest reactivity against mutant p53 or other neoantigens (see Table 2) were subjected to a rapid expansion protocol in which target TILs were incubated with irradiated healthy donor peripheral blood mononuclear cells (PBMC), anti-CD3 (30 ng/mL, clone OKT3, Miltenyi Biotec), and IL2 (3,000 IU/mL) (Aldesleukin, Clinigen) for 14 days. The reactivity against p53 neoantigen in TIL subcultures included in the infusion products for patient 4127 were retrospectively determined, whereas the rest of the patient TILs in Table 2 were prospectively screened.

### Bulk next-generation *TCRB* sequencing

*TCRB* survey or deep sequencing was performed from gDNA by Adaptive Biotechnologies. Frozen, pelleted PBMCs or TILs (5 × 10^4^ to 1 × 10^6^ cells) were submitted for sequencing (see Table 2). Analysis of productive TCR rearrangements was performed using ImmunoSEQ Analyzer 3.0 (Adaptive Biotechnologies).

### Single-cell *TCRA/TCRB* next-generation sequencing

Single-cell TCR sequencing was performed using the Takara SMARTer Human scTCR a/b Profiling Kit – 96 (catalog nos, 634463, 634464, and 640180; Takara Bio) according to the manufacturer’s instructions. Briefly, single cells from populations of interest (i.e.,4–1BB^+^OX40^+^ neoantigen-reactive cells following coculture; also see Coculture of T cells with APCs or tumor cells) were sorted into wells of a 96-well plate and subjected to cDNA synthesis and amplification using SMART technology to incorporate cellular barcoding per the manufacturer’s instructions. cDNA corresponding to *TCRA* and *TCRB* transcripts was further amplified and prepared for sequencing per the manufacturer’s instructions. Sequencing was performed on an Illumina MiSeq instrument with paired-end, 2 × 300-bp reads using the MiSeq Reagent Kit v3 (600 cycle; catalog no. MS-102–3003, Illumina). Read extraction and clonality counts were determined by the MiXCR software package (v.2.1.12, Milaboratory).

### TCR cloning and retroviral T-cell transduction

Reconstructed variable regions of *TCRB* and *TCRA* sequences were linked with a P2A spacer sequence, codon optimized, and cloned into an MSGV1 vector with murine *TCRA/TCRB* constant region-encoding sequences (gene synthesis, cloning, and plasmid extraction by Genscript; ref. 22). Transduction of healthy donor PBLs using fresh viral supernatant was performed as described previously (15). The R175H-TCR used in the treatment of patient 4349 (see Table 1) was previously described (12). Clinical grade GMP retroviral supernatants were obtained from the Vector Production Facility at Cincinnati Children’s Hospital (Cincinnati, OH). Transduction of PBLs from patient 4349 was performed as previously described (23). Transduction efficiency was determined by flow cytometry using an antibody specific for mouse TCRβ (mTCRβ; see Antibodies, flow cytometry, and FACS)

### Antibodies, flow cytometry, and FACS

T cells following a coculture with APCs (see Coculture of T cells with APCs or tumor cells) were stained with antibodies specific for the following human markers: CD4 FITC (clone RPA-T4; 1:20, catalog no. 555346, RRID:AB_395751), OX40 PE (clone ACT35; 1:20, catalog no. 555838, RRID:AB_396161), CD8 PE-cy7 (clone RPA-T8; 1:25, catalog no. 560917, RRID:AB_2033970), 4–1BB APC (clone 4B4–1; 1:20, catalog no. 550890, RRID:AB_398477), and CD3 APC-Cy7 (SK7; 1:25, catalog no. 341090, RRID:AB_400214; all from BD Biosciences). T cells engineered to express a TCR with murine *TCRA/TCRB* constant region sequences were stained with the following antibodies: mTCRβ FITC or BV421 (clone H57–597; 1:20, catalog no. 553170, RRID: AB_394682 or catalog no. 562839, RRID: AB_2737830), CD4 FITC or PE (same as above), OX40 PE (same as above), CD8 PE-cy7 (same as above), and 4–1BB APC (same as above) (all from BD Biosciences). Infusion product T cells were stained with the following antibodies in conjunction with tetramer staining (see Tetramer synthesis): CD3 APC-Cy7, (same as above), CD8 PE-Cy7 (same as above), CD4 BV785 (OKT4; 1:66, catalog no. 317442, RRID:AB_2563242, BioLegend), CD39 FITC (A1; 1:200, catalog no. 328205, RRID:AB_940423, BioLegend), CD69 APC (FN50; 1:25, catalog no. 560711, RRID: AB_1727507, BD Biosciences), and CD62L BV421 (DREG-56; 1:50, catalog no. 304827, RRID:AB_10896429, BioLegend; ref. 24). Analytic flow cytometry was performed on a FACSCanto II, LSRFortessa, or FACSymphony (BD Biosciences) with analysis by FlowJo software (version 10.6.2, TreeStar). All cells were gated via lymphocytes [forward scatter (FSC) and side scatter (SSC)]) and live cells by exclusion of cells stained with propidium iodide (catalog no. P1304MP, Thermo Fisher Scientific; see Supplementary Fig. S2). For TCR isolation 4–1BB^+^ and/or OX40^+^ cells were sorted separately through CD3^+^CD4^+^CD8^−^ (for CD4) and CD3^+^CD4^−^CD8^+^ (for CD8) gates using SH800S or MA900 (Sony Biotechnology). For single-cell transcriptome analysis of patient 4349’s infusion product and PBLs, CD3^+^CD8^+^mTCR^+^ cells (i.e., CD8^+^R175H-TCR^+^ T cells) were sorted using MA900 (Sony Biotechnology).

### Tetramer synthesis

To specifically examine mutant p53–reactive TILs from patients 4266, 4324, and 4350, tetramers were synthesized. We determined minimal epitopes and HLA restrictions for the p53 neoantigen reactivities from these patients as described above (see Coculture of T cells with APCs or tumor cells and Table 1). High-performance liquid chromatography (HPLC)–grade minimal epitope peptides (Genscript) were used in the synthesis of tetramers according to the UV exchange tetramer protocol previously described (24, 25). Briefly, Flex-T A*11:01 UV-exchangeable HLA-monomer was obtained from BioLegend (catalog no. 280007), and A*68:01 and C*06:02 UV-exchangeable HLA-monomers were a generous gift from Dr. Pia Kvistborg (Netherlands Cancer Institute; Amsterdam, Netherlands) for 4350, 4266, and 4324 TILs, respectively. 45 μg of mutant p53 minimal epitopes (containing p53^L111R^, p53^R248W^, p53^T211I^ for patients 4350, 4266, and 4324 TILs, respectively) were coincubated with 10 mg UV-exchangeable biotinylated HLA-monomers in 50 μL 1X PBS for 2 hours on ice under UV-light. Following peptide exchange, 10 μL of fluorescently labeled Streptavidin-PE (catalog no. 405203) and Streptavidin-APC (catalog no. 405207, both from BioLegend) were added to saturation in 2 mL increments on ice 15 minutes each for an hour, centrifuged for 10 minutes at 2,000 rpm, and stored in 4°C until future use.

### ACT of human TYK-nu cancer cells in NSG mice

Animal experiments were approved by the Institutional Animal Care and Use Committees of the NCI and performed in accordance with the NIH guidelines. Six-week-old female NSG mice were obtained from NCI. Two to three million human ovarian cancer TKY-nu cells or 5 million #4259 colon PDX tumor cells in 100 μL of PBS were injected subcutaneously into the right flank area of NSG mice. Following tumor cell injection, the mice were randomized. 2 to 3 weeks after tumor inoculation, 2 or 5 million healthy donor PBLs or 10 or 20 million PBLs from patient 4349 (Supplementary Table S1) that had been genetically engineered with the R175H-TCR, the Y220C-TCR, or were untransduced were intravenously injected in 500 μL of PBS into tumor-bearing NSG mice. Three daily doses of 180,000 IU of recombinant human IL2 (Aldesleukin, Clinigen) in 500 μL of PBS were injected intraperitoneally following the T-cell transfer. Tumor growth was measured twice a week, and tumor size was calculated as the product of two perpendicular measurements. All experiments were conducted in a blinded manner.

Sample sizes of 4, 5, or 10 mice per experimental arm were used to ensure adequate power. Two-way ANOVA was used for statistical analysis of tumor growth between experimental arms using Prism 8. Log–rank test was performed for statistical analysis of mouse survival using Prism 8.

### IHC and RNAscope analysis of patient 4349’s biopsies

Skin biopsy specimens for pathological analysis were obtained at the time of patient 4349’s treatment, and 6 days and 209 days after the treatment. These were fixed in 4% paraformaldehyde (catalog no. J61899.AK, Thermo Fisher Scientific) overnight, transferred to 70% ethanol, and embedded in paraffin. All IHC staining on anatomic pathology surgical specimens was performed in the NCI Center for Cancer Research, Laboratory of Pathology, a laboratory approved by the College of American Pathologists Laboratory Accreditation Program. The following antibodies were clinically validated to the satisfaction of the College of American Pathologists checklists and inspectors, and their specificities were regularly checked and maintained by the Laboratory of Pathology: CD3 (clone 2GV6; prediluted; Ventana), CD4 (clone SP35; prediluted; Roche), CD8 (clone SP57; prediluted; Roche), PD-1 (clone NAT105; prediluted; Roche), PD-L1 (clone SP142; prediluted; Abcam), pan-HLA class I (clone HC-10; 1:1000; in-house), HLA-DR (clone TAL.1B5; 1:200; Dako), p53 (clone DO-7; 1:1000; Dako). RNAscope against MSGV1 to detect R175H-TCR^+^ T cells *in situ* was performed according to the manufacturer’s instructions (323900, ACD; a pair of probes was designed from following sequence: AGTCTGGAGACCTCTGGCGGCAGCCTACCAAGAACAACTGGA). Detection of HLA-A*02:01 by RNAscope was performed using a pair of probes designed from base pairs 622–659 of the HLA-A*02:01 DNA sequence.

### Flow cytometry–based multiplex blood serum cytokine analysis

Serum samples were collected from patient 4349. The levels of 13 proteins, including IFNγ, IL6, and IL10, were analyzed using LEGENDplex CD8/NK panel (catalog no. 740267, BioLegend) according to the manufacturer’s instructions by flow cytometry.

### Sample preparation and sequencing for single-cell transcriptome analysis

CD8^+^R175H-TCR^+^ T cells in patient 4349’s infusion product (RX) and the PBLs obtained 6 weeks after ACT (PBL_6w) were sorted (see Antibodies, flow cytometry, and FACS), resuspended in PBS at 5 × 10^5^ cells/mL, and loaded onto a Chromium Controller (10X Genomics) for single-cell sample preparation. Two channels per reaction were used to prepare each sample for sequencing following the manufacturer’s protocol. Briefly, 10,000 T cells per channel were loaded on the Chromium Controller with the targeted cell recovery of 6,000 single cells. The single-cell cDNA samples were first universally amplified by a 16-cycle PCR reaction using a thermocycler (T100, Bio-Rad) and the Chromiun Next GEM Single Cell 5′ Reagent Kits V2 (catalog no. PN-1000265, 10X Genomics) according to the manufacturer’s instructions. cDNAs for TCR (VDJ) sequencing were further enriched by two additional PCR reactions using TCR-specific primers according to the manufacturer’s protocols (catalog no. PN-1000252, 10X Genomics). The whole transcriptomes (GEX) from the same cDNA samples were amplified after cDNA fragmentation per the manufacture’s protocol. The processed single-cell cDNA samples were sequenced using an Illumina NextSeq 550 sequencer (High Output Kit v2.5; Read1: 26 b.p; Read2: 98 b.p). The GEX libraries were sequenced using the Illumina NextSeq 2000-P3 kit (Read 1: 26 b.p.; Read 2: 90 b.p.).

### Bioinformatic analysis for single-cell transcriptome sequencing data

Single-cell transcriptome sequencing data were first processed using Cell Ranger pipelines (v3.1.0; 10X Genomics). The demultiplexed sequencing data were mapped to the reference genome (human reference GRCh38–2020-A, 10X Genomics). Gene expression matrices were generated from the unique molecular identifier-collapsed read counts on individual cell barcodes. Gene expression matrices with error-corrected hdf5 reads and cell barcode included cells and annotated genes. Low-quality cells or doublets were filtered out based on the feature distribution, and genes with fewer than five read counts across all cells were removed. The expression matrix was converted to transcripts per million (TPM) using R (3.6.3). Further analyses were performed using R package Seurat v4 (24, 26). A common gene expression matrix was generated for patient 4349’s infusion product and 6-week-posttreatment PBLs. The expression matrix was standardized by regressing out highly variable genes and mitochondrial genes followed by normalization and scaling. We excluded all *TRAV*/*TRBV* genes to remove endogenous TCR expression as a source of clustering bias prior to processing by Seurat. Principal components (PC) were generated, and elbow and Jackstraw plots were used to define significant PCs for clustering. Uniform Manifold Approximation and Projection (UMAP) plots were generated by the clustered PCs. Cluster markers were obtained for individual clusters using default parameters. Heatmap of genes of interest were generated by DoHeatmap function using a downsample parameter of 100. The featureplot function was used to display genes of interest on the UMAP. Single-cell gene set enrichment analysis (scGSEA), a rank-based gene signature metric that computes the expression score of a gene list relative to all other genes using single-cell RNA (scRNA) expression, was performed on patient RX and PBL_6w single-cell transcriptome data as described before (24, 27). In brief, normalized scRNA gene expression matrices with barcodes were used as input in conjunction with 72 single cell gene set lists or gene sets from the bulk RNA sequencing (496 gene signatures from ImmunesigDB). Only CD8^+^ cell signatures were considered. scGSEA scores for all gene signatures were calculated using the gene set variation analysis (GSVA) function and z-scaled for cross-signature comparisons across cells and samples. Clustered correlation matrices between various gene signatures from single T cells were generated using the R package Corrplot (https://github.com/taiyun/corrplot). Cell types with highest correlation scores with cluster markers generated using patient RX and PBL_6w in conjunction with differentially expressed genes were used to assign cell-types to the RX and PBL_6w cells.

### Data availability

Single-cell transcriptome data for patient 4349’s RX and PBL_6w samples and WES/RNA-seq data for 37 patient tumors in Supplementary Table S1 are available in dbGap (https://www.ncbi.nlm.nih.gov/gap/) under accession number phs002928.v1. WES/RNA-seq data for the 41 samples that were previously described (5, 10, 12, 15, 16) are available in dbGaP under accession number phs001003.v2.p1. All other data supporting the findings of this study are available within the manuscript and its supplementary data files or from the corresponding author upon reasonable request. The publicly available single-cell transcriptome datasets that were used in this study are listed in Supplementary Tables S2, S3, and S4.

### Generation of Fig. 1A

**Figure 1.**
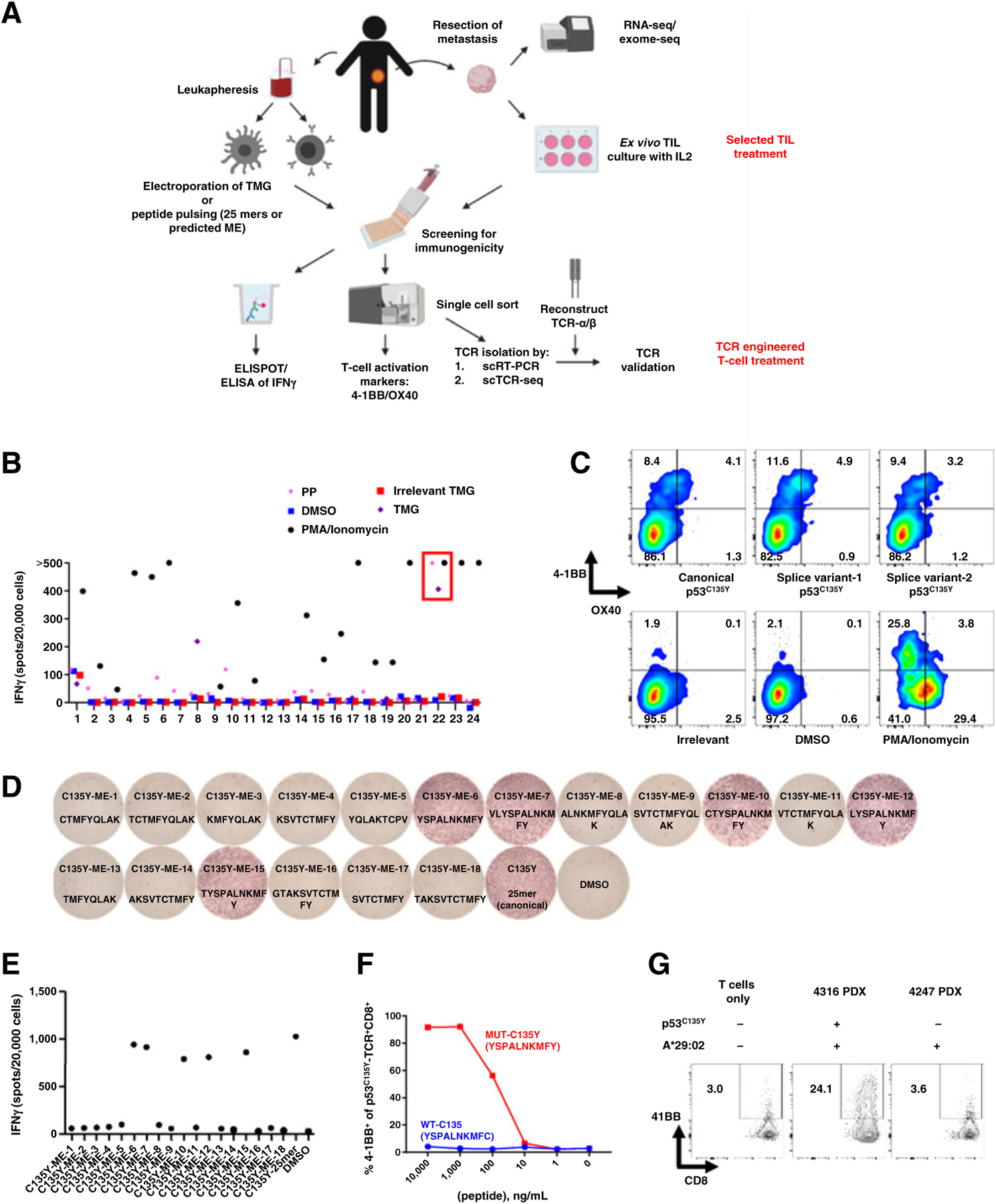
Unbiased neoantigen screening of TILs from patient 4316 identifies TILs/TCR reactive with mutant p53^C135Y^. **A,** Diagram depicting the unbiased neoantigen screening for immunogenicity of shared *TP53* mutations. **B,** ELISpot assay measuring IFNg secretion in TILs. The 24 TIL fragment subcultures from colorectal cancer patient 4316 were screened against the somatic mutations identified in the patient’s tumor, including p53^C135Y^. TIL fragment 22 showing increased IFNγ secretion against TMGs and PPs that included p53^C135Y^ is highlighted in red (*n* = 1). **C,** Flow cytometric analysis of cell surface 4–1BB/OX40 expression upon parsing individual reactivities of TIL fragment 22. An irrelevant peptide (KIAA1328^K386R^) and DMSO (vehicle) as negative controls and PMA/ionomycin as a positive control are included (*n* = 1). **D,** Functional determination of MEs for TCR-B by ELISpot measurement of IFNγ secretion. Eighteen candidate MEs predicted to bind patient’s class I HLAs were tested. The amino acid sequences of the tested peptides are listed (*n* = 1). **E,** Quantification of IFNγ spots from panel (**D**). **F,** Functional testing of avidity of TCR-B. Avidity was determined by coculturing TCR-expressing healthy donor PBLs with autologous imDCs pulsed with serially diluted ME6. 4–1BB expression was measured by flow cytometry (*n* = 1). **G,** Autologous tumor cell recognition by 4316 TCR-B. The 4316 autologous PDX was established into a cell line and was cocultured with TCR-B–expressing PBLs. 4–1BB upregulation in p53^C135Y^-TCR^+^CD8^+^ cells is shown. The 4247 PDX line with matching HLA but lacking a p53^C135Y^ mutation was included as a negative control. (*n* = 1) Experiments in (**B**) to (**G**) were independently repeated once. seq, sequencing. PP, peptide pool.

The schematic of neoantigen identification and TCR isolation in Fig. 1A was created using BioRender.com.

## Results

### Unbiased screening identifies TILs/TCRs recognizing p53 neoantigens in patients with epithelial cancers

We screened tumor samples from 163 patients with chemorefractory metastatic epithelial cancers enrolled in a tissue procurement protocol (NCT00068003) for clinical trials of ACT (NCT01174121 and NCT03412877) to generate a library of neoantigens encoded by shared *TP53* mutations. Fifty-nine percent of tumor samples (97/163 patients) harbored nonsynonymous *TP53* mutations (Supplementary Fig. S3A). Samples with nonsense *TP53* mutations or WT *TP53* were excluded from this study. Among the screened *TP53* mutations, missense mutations were most common (80%; Supplementary Table S1), followed by frameshifts (19%) and in-frame insertions (1%; Supplementary Fig. S3A). Among the samples containing missense *TP53* mutations, those that occurred at 1% frequency or greater were considered “hotspot” mutations (49%), and the remaining 51% were considered “non-hotspot” mutations (Supplementary Fig. S3B; ref. 28). Both the “hotspot” mutations and the “non-hotspot” *TP53* mutations in our patient cohort were highly clonal based on WES (Supplementary Fig. S3C). This was expected given the likelihood that many or all of these *TP53* mutations represented driver mutations that were critical for maintaining tumorigenicity (29), and simultaneously provided a rationale for targeting these mutations rather than subclonal mutations, which could potentially lead to tumor immune escape.

A schematic of *TP53* neoantigen identification and TCR isolation from TILs is presented in Fig. 1A. As an example, we describe the identification of “non-hotspot” p53^C135Y^-reactive TILs and TCRs from lung metastases of a patient with metastatic colorectal cancer (patient identifier 4316 in Supplementary Table S1). WES identified 158 mutation targets for which TMG-RNAs and 25-mer peptides were synthesized for *in vitro* screening of the 24 TIL subcultures. TIL subculture F22 showed increased secretion of IFNγ in response to TMG-RNA and peptide pools that contained the p53^C135Y^ peptide (Fig. 1B). When the individual peptides in the pool were tested, three p53^C135Y^ peptides, including the canonical form and two splice variants, led to upregulation of T-cell activation makers 4–1BB and OX40 relative to the DMSO or the irrelevant peptide control (Fig. 1C). Candidate TCR sequences were isolated from the 4–1BB^+^OX40^+^ T cells, reconstructed, and retrovirally transduced into healthy donor PBLs. For one candidate TCR isolated from CD8^+^ T cells (TCR-B), 18 candidate minimal epitope (ME) sequences were selected for testing. TCR-B–transduced T cells upregulated IFNγ secretion against ME6 and 4 other peptides that shared the same core sequence as ME6 (Fig. 1D and E), and therefore ME6 was subsequently used for testing the avidity and specificity of TCR-B. Against serially diluted peptides, TCR-B showed strong avidity for the mutant peptide without reacting with the WT peptide (Fig. 1F). Next, it was determined that the TCR-B–transduced T cells secreted IFNγ in the context of HLA-A*29:02 (Supplementary Fig. S3D and S3E). Finally, the ability of this TCR to recognize tumor cells was tested, and it was shown that the TCR-B^+^ PBLs upregulated 4–1BB when cultured with autologous PDX tumor cells expressing the p53^C135Y^ neoepitope but not control tumor cells lacking this mutant p53 neoepitope but with a matching HLA (Fig. 1G).

By iterating this approach for the additional 77 samples, we identified a total of 21 unique TIL reactivities (27%). Eight had been previously reported (10–12, 15,16) and 13 were new reactivities, and two different patient TILs reacted against the same *TP53* mutation (p53^R175H^ and p53^Y220C^) with the same HLA restriction (Table 1). Redundancies of TCRs recognizing the same mutations led us to identify a total of 39 TCRs, including 16 HLA class I–restricted TCRs and 23 HLA class II–restricted TCRs (Supplementary Table S5). All the TCRs listed in the library were validated to react with the mutant peptides, although some of the TCRs showed WT peptide recognition at super-physiologic levels of peptide concentration (Supplementary Fig. S4). Because some TCRs targeting “hotspot” mutations, including R175H, Y220C, and R273C, were paired with common HLAs, such as A*02:01 and DPB1*04:02, they could potentially be used to treat a broad range of patients. Overall, approximately 7.3% of patients with solid cancers are estimated to share the *TP53* mutations and HLAs as the TCRs listed in the library (Table 1).

### Antigen and HLA-specific target recognition by mutant p53-reactive TCRs

**Figure 2.**
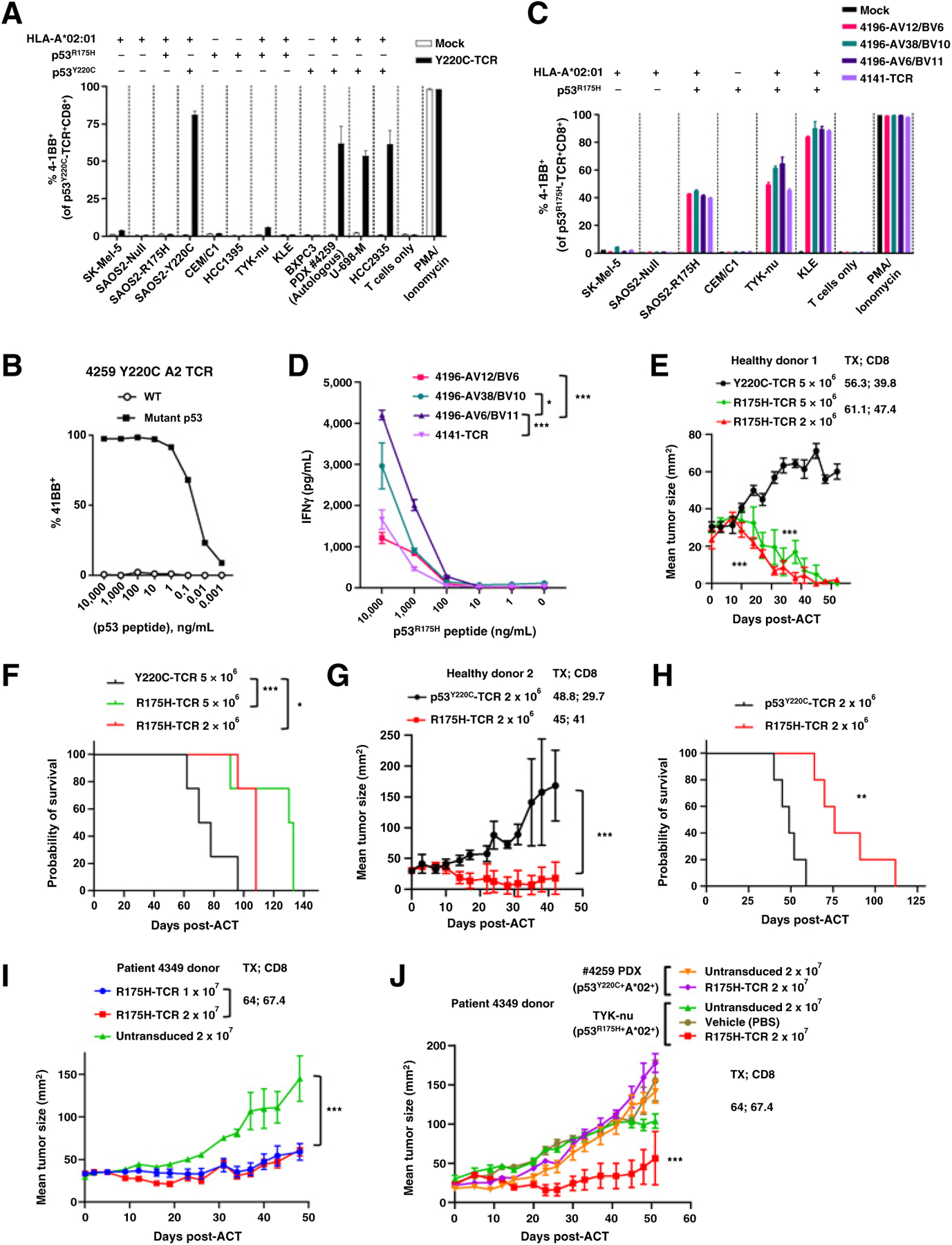
Characterization of mutant p53–reactive TCRs. **A,** Tumor-cell recognition by p53^Y220C^-TCR (Y220C-TCR). Y220C-TCR was expressed in healthy donor PBLs and was tested against a panel of tumor cell lines with different *TP53* mutations and HLAs. Following coculture with tumor cell lines, cell surface upregulation of 4–1BB on the T cells was measured by flow cytometry. Mock transduced T cells were included as a negative control (mean ± SEM, *n* = 3). **B,** Titration curve showing the avidity of Y220C-TCR against theWT and mutant Y220C ME. 4–1BB upregulation in healthy donor PBL transduced with Y220C-TCR following coculture with A*02:01^+^ T2 cells pulsed with the serially diluted ME wasmeasured by flow cytometry (*n* = 1). **C**, Comparison of 4 HLA-A*02:01-restricted TCRs targeting p53^R175H^ based on tumor cell reactivity. 4–1BB upregulation in p53^R175H^-TCR^+^CD8^+^ cells was measured by flow cytometry (mean ± SEM, *n* = 2). **D,** Titration curves for 4 TCRs targeting p53^R175H^. HLA-A*02:01^+^ T2 cells were pulsed with the serially diluted p53^R175H^ ME and cocultured with TCR-transduced T cells. IFNg secretionwas measured by ELISA (mean ± SEM, *n* = 3). Preclinical ACT of NSG mice bearing TYK-nu cancer cells using the R175H-TCR (4196-AV6/BV11)-engineered human PBL. Tumor size was calculated as the product of two perpendicular measurements. Tumor measurement was discontinued when the first mouse was euthanized [mean ± SEM, *n* = 4 (**E** and **F**), 5 (**G**, **H**, and **J**), and 10 (**I**)]. Donor information, transduction efficiency (TX; %), and CD8^+^ cell frequency (%) are given. **E** and **G,** Tumor growth following ACT of two different healthy donor PBLs transduced with the R175H-TCR or the irrelevant Y220C-TCR. **I** and **J,** Tumor growth following ACT of patient 4349’s PBL (1 or 2 × 10^7^ cells) transduced with the R175H-TCR or untransduced (2 × 10^7^ cells). **J,** Mice injected with either TYK-nu cells or the control 4259 PDX cells were treated with untransduced T cells, R175H-TCR–engineered T cells, or vehicle (PBS). Statistical analyses by two-way ANOVA (**D**, **E**, **G**, **I, J**) and by log-rank tests (**F** and **H**). *, *P* < 0.05; **, *P* < 0.01; ***, *P* < 0.001. **A**–**D** were independently repeated at least once.

To further characterize the mutant p53–reactive TCRs in the library, we first explored whether T cells transduced with a TCR targeting the HLA-A*02:01–restricted “hotspot” p53^Y220C^ neoepitope (referred to as Y220C-TCR) could recognize allogeneic and autologous tumor cells. When cocultured with the cancer cells, healthy donor T cells expressing Y220C-TCR specifically upregulated 4–1BB in the presence of cells that were positive for both p53^Y220C^ and A*02:01 (Fig. 2A) and in the presence of A*02:01^+^ T2 cells pulsed with the Y220C ME but not the WT ME (Fig. 2B). The remaining HLA class I–restricted TCRs with available autologous or allogeneic tumor cells also recognized the tumor cells in an antigen- and HLA-specific manner (TCRs from patients 4141, 4196, 4266, 4324, and 4350; Table 1; Fig. 2C; and Supplementary Fig. S5). The TCR targeting p53^Y220C^ with HLA DRB1*04:01–restriction did not react with native autologous PDX cells, but expression of HLA DRA1/DRB1*04:01 rendered the tumor cells recognizable (Supplementary Fig. S5C). In line with these efforts, 4 HLA-A*02:01–restricted TCRs targeting p53^R175H^ were functionally compared using a panel of cell lines. For all four TCRs, T cells specifically upregulated 4–1BB when cultured with SAOS2-R175H, TYK-nu, and KLE cells, which are all p53^R175H+^ and HLA-A*02:01^+^, but not when cultured with control tumor cells that were singly positive for p53^R175H^ or HLA-A*02:01 (Fig. 2C). Although 4–1BB upregulation on CD8^+^ cells cultured with p53^R175H+^HLA-A*02:01^+^ tumor cells was similar between the four TCRs, T cells expressing the 4196 AV6/BV11 TCR secreted the highest level of IFNγ when cocultured with T2 cells pulsed with the serially diluted mutant peptide (Fig. 2D) and this TCR was subjected to further preclinical testing (hereafter, referred to as R175H-TCR).

TYK-nu cells successfully grew in NSG mice but engraftment of KLE cells was not observed. We evaluated the efficacy of ACT using PBLs transduced with R175H-TCR against engrafted TYK-nu cells. First, we tested this TCR by transducing PBLs from two different healthy donors. ACT with the healthy donor PBLs (2/5 × 10^6^ cells) expressing the R175H-TCR led to tumor regression and improved overall survival relative to the control-TCR transduced PBLs (Y220C-TCR) (Fig. 2E–H). PBLs from a patient transduced with R175H-TCR also inhibited tumor growth and showed specificity for p53^R175H+^ TYK-nu cells compared with control p53^Y220C+^ 4259 PDX (Fig. 2I and J).

### Treatment of patients with ACT using autologous TILs targeting p53 neoantigens

Next, we tested the feasibility, safety, and efficacy of ACT targeting p53 neoantigens in 12 patients with chemorefractory epithelial cancers by transferring *ex vivo*–expanded TILs that contained varying numbers of T cells naturally reactive with mutant p53 (Table 2; Supplementary Fig. S1). The *TP53* mutations that were targeted included both “hotspot” and “non-hotspot” mutations. Four and seven patient infusion products contained HLA class I–restricted and HLA class II–restricted mutant–reactive p53 TILs, respectively, and one infusion product contained two different TIL reactivities with each restriction. Two patients (4127 and 4343) showed a partial response (PR) by RECIST 1.0 criteria with response durations of 4 and 6 months, respectively (Supplementary Fig. S6; ref. 16).

**Figure 3.**
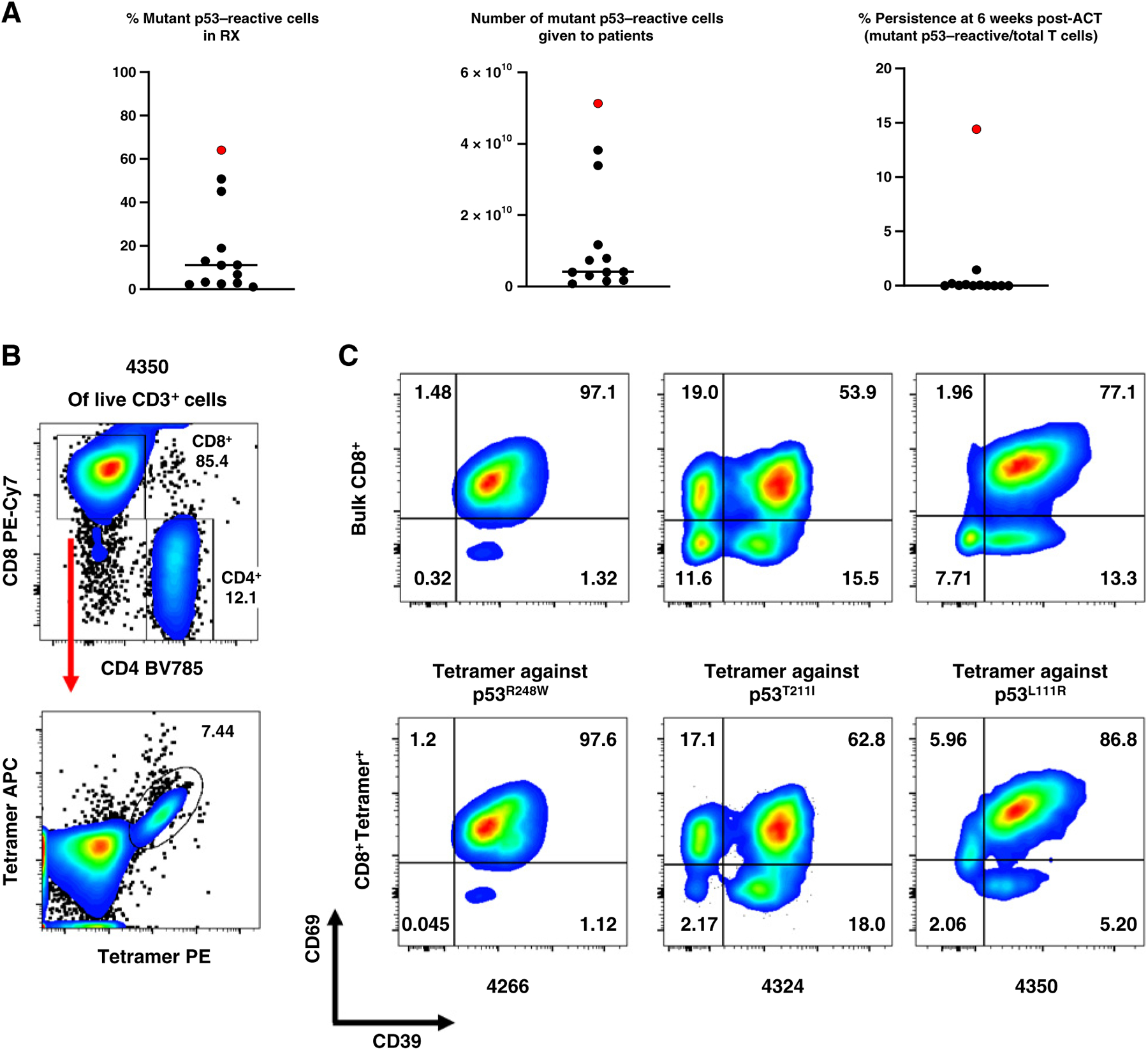
Autologous TIL ACT for the treatment of 12 patients with chemorefractory epithelial cancers. **A,** Characterization of the infusion products for the 12 autologous TIL ACTs. The ACT sample with genetically engineered PBL for patient 4349 is included for comparison and is marked in red. Bar denotes median. Detailed information is available in Table 2. RX, infusion product. **B,** Representative tetramer staining analysis by flow cytometry. Following positive gating of live CD3^+^ cells of patient 4350’s infusion product TILs, CD4, and CD8 gating (top) and tetramer staining of CD8^+^ cells (bottom) are presented. **C,** Phenotypic analysis of antigen-specific or bulk CD8^+^ T cells from the infusion products for patients 4266, 4324, and 4350 by flow cytometry. Expression of CD39 and CD69 in Bulk CD8^+^ T cells (CD3^+^CD8^+^; top) or tetramer-stained cells (CD3^+^CD8^+^Tetramer^+^; bottom) are shown. Due to sample limitations, **A**–**C,** were not independently repeated.

After a preliminary analysis of TIL infusion products demonstrated elevated levels of PD-1 expression, a pretransfer dose of pembrolizumab was incorporated into the clinical protocol to prevent inhibition of the transferred cells (ref. 4; Table 2). The median frequency of mutant p53–reactive TILs in the infusion products determined by deep sequencing of *CDR3B* was low (median 8.9%; range, 1%–50.8%; Fig. 3A). For the 2 patients who had a PR (4127 and 4343), 2.8% (4127) and 1% (4343) of their infusion products were mutant p53–reactive cells. The infusion products for patients 4114 and 4343 contained additional neoantigen-reactive cells (Table 2; Supplementary Table S6). For patient 4343 who had a PR, it would be difficult to ascertain precisely what contribution the mutant p53 reactivity might have had in their response (16). The 12 patients received a median of 8.1 × 10^10^ autologous bulk TILs or 4.0 × 10^9^ mutant p53–reactive cells (range, 7.7 × 10^8^–5.2 × 10^10^) per patient (Fig. 3A). The median persistence of mutant p53–reactive cells at 6 weeks posttreatment was 0.01% (range, 0%–1.45%; Fig. 3A). The phenotype of the infused TILs showed a high degree of exhaustion: A median of 43%, 33%, and 93% of the bulk TILs were positive for PD-1, TIM3, and CD39, respectively (Table 2). Conversely median CD62 L expression, a marker for less differentiated naïve and central memory T cells, was 5.02% (range, 0.89%–29.24%; Table 2). In one study, it was demonstrated that CD39^−^CD69^−^ T cells with stem-like properties were associated with complete melanoma regression but CD39^+^CD69^+^ T cells showed a differentiated phenotype and were associated with poor TIL persistence (24). We specifically interrogated the differentiation phenotype of mutant p53–reactive TILs based on CD39 and CD69 expression using HLA class I tetramers, given their low and variable frequencies within the infusion products (Fig. 3B): Among the patients who received HLA class I–restricted mutant p53–reactive TILs, the infusion products for patients 4266, 4324, and 4350 that had HLA-A*68:01, C*06:02, and A*11:01 restriction, respectively, were chosen based on the availability of tetramers and infusion products. The tetramer^+^CD8^+^ TILs in the infusion products for patients 4266, 4324, and 4350 consisted mostly of differentiated CD39^+^CD69^+^ T cells (97.6, 62.8 and 86.8%, respectively) and very few stem-like CD39^−^CD69^−^ T cells (0.045, 2.17, and 2.06%, respectively; Fig. 3C).

### TCR-engineered PBL have improved frequency, phenotype, and persistence

TILs generally show more differentiated phenotypes than PBLs due in part to constant exposure to tumor antigens and the suppressive environment (30–33). Therefore, we tested whether PBL engineered to express a mutant p53–reactive TCR would have improved differentiation/exhaustion phenotypes and *in vivo* persistence relative to TILs. We engineered autologous PBL from patient 4349 with an allogeneic p53–reactive TCR for experimental treatment of chemorefractory breast cancer. The 48-year-old HLA-A*02:01^+^ patient, who had progressed through 10 prior lines of hormonal and chemotherapies (Supplementary Table S7), was treated with autologous PBLs engineered with R175H-TCR, which had shown antitumor efficacy in the murine model (Fig. 2E–J).

**Figure 4.**
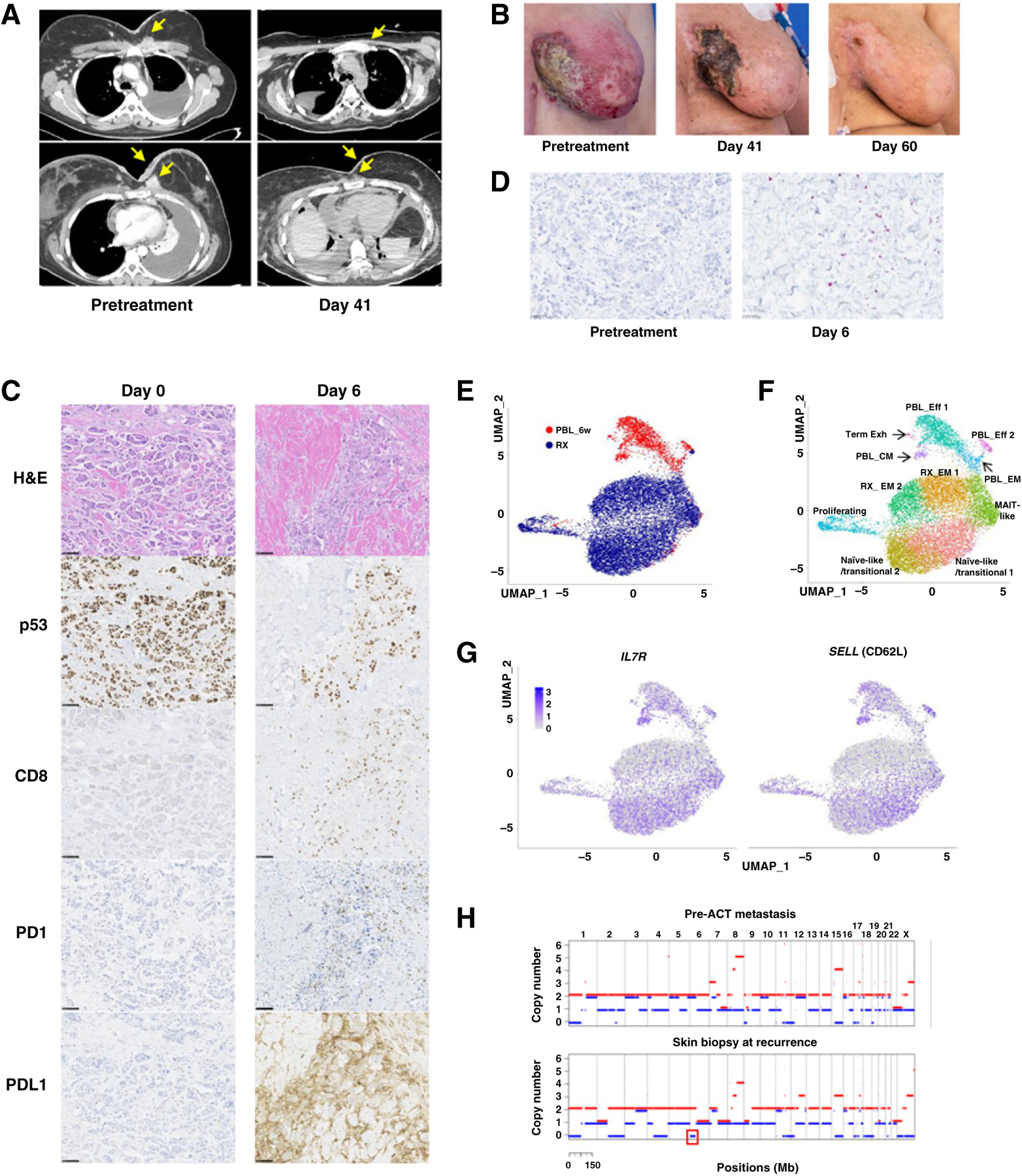
ACT with R175H-TCR–engineered autologous PBLs for the treatment of patient 4349 with chemorefractory breast cancer. **A,** Contrast-enhanced CT scans of the chest of patient 4349 before (left) and day 41 after the infusion of 5.3 × 10^10^ R175H-TCR–expressing PBLs (right). **B,** Pictures showing changes in metastatic skin deposits before the cell therapy (left), at day 41 (middle), and day 60 (right). **C,**) IHC analysis of the skin biopsies at day 0 before the cell therapy (left) and at day 6 (right). Scale bar, 100 μm. Following ACT, a decrease in tumor cell numbers and p53^+^ tumor cells and an increase in CD8^+^ T cells, PD-1^+^ T cells, and PD-L1^+^ tumor cells were detected. **D,** Detection of R175H-TCR^+^ T cells by RNAscope. Tumor-infiltrating T cells that expressed the R175H-TCR are visualized using an RNAscope probe against the MSGV1 3′ UTR. Each purple dot represents one RNA molecule. **E**–**F,** UMAP projection of 12,993 R175H-TCR^+^CD8^+^ T cells: 10,893 infusion product T cells (RX) and 2,100 6-week posttreatment PBLs (PBL_6w). **E,** UMAP clustering of single cells indicated by the sample source. **F,** Clustering based on whole transcriptome analysis. Phenotypic clusters are represented using different colors. **G,** Expression of indicated genes overlaid on the UMAP projection of RX cells and PBL_6w cells. **H,** Copy-number analysis of patient 4349’s pre-ACT metastasis tumor fragment 6 (top) and the skin biopsy at recurrence (bottom) by WES. LOH of chromosome 6 containing HLA-A*02:01 is highlighted in a red box. H&E, hematoxylin and eosin; Eff, effector T cells; EM, effector memory T cells; CM, central memory T cells; Term Exh, terminally exhausted T cells; MAIT, mucosa-associated invariant T cells.

WES of a lymph node metastasis from the patient had identified 119 nonsynonymous somatic mutations, including p53^R175H^, but no mutant p53^R175H^–reactive TILs were detected (16). The frequency of R175H-TCR^+^ cells in the infusion product following retroviral transduction and rapid expansion was 64%, which was higher than that of any of the selected TIL infusion products (Fig. 3A; Table 2). The patient presented with advanced disease at the time of treatment: fungating masses that had eroded the tissue of bilateral breasts, a left pleural effusion, and cardiac tamponade requiring a surgical pericardial window 6 days before the ACT treatment. The patient received a standard lymphodepleting chemotherapy followed by ACT of 5.3 × 10^10^ transduced cells with no postinfusion IL2 due to the severely compromising cardiopulmonary disease. Upon cell infusion, the patient developed grade 3 acute cytokine release syndrome (CRS; ref. 34), leading to respiratory distress requiring intubation within minutes of cell infusion and hypotension requiring two vasopressors within hours of infusion (Supplementary Fig. S7A). The day after cell infusion, the patient received one dose of methylprednisolone (90 mg). Within hours of receiving steroids the patient’s hypotension resolved, and she was weaned from the ventilator. At follow-ups 6 and 14 weeks post–cell therapy, target lesions were down 37% and 55%, respectively by RECIST 1.1 criteria (Fig 4A). The metastases in the pericardium, chest wall, and the subcutaneous tumor deposits significantly decreased and all detectable skin lesions completely resolved by day 60 post–cell therapy (Fig. 4A and B).

The genetically engineered infusion product for patient 4349 contained higher numbers of stem-like CD39^−^CD69^−^ cells (32.7%) relative to the TILs from the naturally selected patients with TIL, 4266 (0.045%), 4324 (2.17%), or 4350 (2.06%) (Fig. 3C; Supplementary Fig. S7B). Immunostaining of the skin biopsies at day 0 before ACT but after lymphodepleting chemotherapy revealed healthy tumor cells with high expression of p53 (Fig. 4C). At day 0, we did not detect any tumor-infiltrating CD8^+^ T cells, PD1^+^ T cells, or PDL1^+^ tumor cells. However, at day 6 post-cell therapy, we saw necrotic tumor cells, a decrease in p53^+^ tumor cells, and a dramatic increase in CD8^+^ T cells as well as PD1^+^ T cells and PDL1^+^ tumor cells. Targeting the 3′ UTR sequence of the MSGV1 retroviral backbone used to express the R175H-TCR by RNAscope, we determined that many tumor-infiltrating T cells were MSGV1^+^ cells expressing the R175H-TCR (Fig. 4D). In addition to the increase in PD1^+^ T cells in the skin biopsies, we detected increased PD1^+^R175H-TCR^+^ T cells in the peripheral blood and pleural effusion at day 14 (Supplementary Fig. S7C), and the patient received one dose of pembrolizumab at day 16. 12 hours later, the patient experienced fever, respiratory distress, kidney dysfunction requiring dialysis, and a maculopapular rash pathologically confirmed as neutrophilic folliculitis and perivascular neutrophilic inflammation. The rash resolved without further therapy and did not result in any scarring or permanent damage. Kidney dysfunction naturally resolved, and the patient no longer required dialysis at 2 weeks post-pembrolizumab treatment. The symptoms coincided with a sharp increase in the serum cytokine levels of IFNγ, IL6, and IL10 (Supplementary Fig. S7A), and the patient did not receive additional pembrolizumab doses due to the adverse symptoms.

Persistence of R175H-TCR^+^ T cells at 6 weeks post-ACT was higher than that seen in any of the patients receiving TIL treatments (Fig. 3A; Supplementary Fig. S7D). To further delineate the phenotype of the R175H-TCR^+^ T cells following ACT, we performed single-cell whole transcriptome analysis on the RX and the PBL_6w: a total of 10,873 and 2,100 R175H-TCR^+^CD8^+^ T cells were sorted and analyzed from the RX and the PBL_6w samples, respectively. The RX cells and PBL_6w cells clustered separately when projected by UMAP (Fig. 4E). According to differentially expressed genes, gene expression signatures, and correlations with other published datasets (24, 35–41), 11 clusters were identified (Fig. 4F; Supplementary Table S2, S3, and S4). Although there were clusters of cells in both RX and PBL_6w that expressed markers of exhaustion, both RX and PBL_6w samples contained clusters that expressed genes associated with naïve or memory phenotypes (Fig. 4F–G; Supplementary Fig. S8A–S8B). The PBL_CM cluster expressed hallmark genes associated with memory and stem-like phenotypes, such as *SELL* (CD62L), *IL7R, TCF7* (TCF1), and *LEF1,* and had positive correlations with other datasets derived from stem-like T cells (24) or central memory cells (ref. 36; Fig. 4G; Supplementary Fig. S8A–S8B; Supplementary Tables S2 and S4). In line with these data, we detected functional R175H-TCR^+^ T cells secreting IFNγ upon coculture with imDC pulsed with the p53^R175H^ ME from the PBL collected at 4 months post-ACT, reinforcing the notion that circulating R175H-TCR^+^ memory T cells were present (Supplementary Fig. S8C).

At 6 months post-ACT, the patient progressed with new cutaneous metastases on the bilateral breasts. A skin biopsy confirmed the presence of tumor cells with intact expression of p53 and pan-HLA class I, as well as a lack of R175H-TCR^+^ T cells (Supplementary Fig. S9A). However, WES of the biopsy revealed that a portion of chromosome 6 was lost, resulting in HLA LOH, including HLA-A*02:01 (Fig. 4H; Supplementary Fig. S9B). We did not detect HLA LOH from the two metastases we had originally resected to generate TILs (Supplementary Fig. S9B–S9C). In addition, we orthogonally validated the loss of HLA-A*02:01 in the progressing lesion by RNAscope. In contrast to high expression of HLA-A*02:01 in the tumor cells in the pretreatment biopsy, the vast majority of the tumor cells in the progressing lesion did not express HLA-A*02:01 (Supplementary Fig. S10). The patient died at 8 months post-ACT due to complications secondary to disease progression.

## Discussion

Despite recent advances in targeted cancer therapies, *TP53* mutations, the most frequent mutations in cancer, remains “undruggable” (42). ACT may provide a unique opportunity to target *TP53* mutations but its effectiveness against *TP53* mutations has not been systematically tested. In this study, we describe a library of TCRs targeting shared *TP53* mutations, including both “hotspot” and “non-hotspot” *TP53* mutations. These well-defined TCRs can be readily available to transduce patient’s PBL to target p53-mutated cancers in the same manner as chimeric antigen receptor (CAR) T cells are produced; therefore, this approach is expected to reduce the time required for neoantigen screening and extensive cultures of T cells, which can take several months to a year. Furthermore, the broad applicability of the mutant p53–reactive TCR library contrasts with targeting private neoantigens, which are only applicable for a single autologous treatment (5, 6). We are making the full sequences of the 39 TCRs available (Supplementary Table S5) and are continuing to add new TCRs to the library. Some of the mutant p53–reactive TCRs reacted with autologous or allogeneic cancer cells in a *TP53* mutation and HLA-specific manner. Notably, ACT of the T cells engineered to express R175H-TCR led to a significant decrease in the growth of TYK-nu cancer cells in NSG mice. TYK-nu cells have been shown to have low cell surface expression of the p53^R175H^ epitope:HLA-A*02:01 complex (1.5 copies/cell) (14); however, the R175H-TCR–engineered PBLs effectively targeted the tumor cells *in vitro* and *in vivo*. Various studies have shown that CD4^+^ T cells can be efficacious in treating cancers in both preclinical and clinical settings (1, 43–46). For example, Tran and colleagues reported a case study where a patient with metastatic cholangiocarcinoma showed a complete response following TIL treatment consisting of CD4^+^ autologous TILs targeting a mutated ERBB2IP epitope (1). In general, however, because tumor cells do not normally express HLA class II, the CD4^+^ TILs or TCRs are not expected to directly recognize tumor cells. For instance, the TCR targeting p53^Y220C^ with DRB1*04:01 restriction only recognized autologous tumor cells that were transduced with DRA1/DRB1*04:01 (Supplementary Fig. S5C). Because APCs may be required for CD4^+^ T cells to indirectly recognize tumor antigens, humanized mouse xenograft models with intact human APCs may be useful to further study the *in vivo* efficacy of ACT of CD4^+^ TCRs targeting p53 mutations.

Our analysis of the 12 infusion products used for autologous TIL treatment identified the limitations of naturally selecting mutant p53–reactive TILs, including low frequencies of mutant p53–reactive T cells, exhausted/differentiated immunophenotypes, and a lack of persistence of the infused T cells. Chronic antigen exposure and/or an immunosuppressive tumor microenvironment may contribute to the exhausted/differentiated phenotypes of TILs, which in turn may limit the proliferative potential and persistence of TILs postinfusion (47). For instance, approximately 98% of p53^R248W^-reactive cells in the infusion product for patient 4266 exhibited an exhausted/differentiated phenotype of CD39^+^CD69^+^. Despite the large number of p53^R248W^-reactive cells (5.3 × 10^10^ cells) given to patient 4266, we detected very few in circulation at 6 weeks postinfusion (Table 2). In contrast, PBLs genetically engineered with TCRs showed improved persistence. In addition, our single-cell whole transcriptome analysis of patient 4349’s TCR-engineered PBLs at 6 weeks post-ACT demonstrated that a cluster of T cells had acquired a central memory phenotype with features of stem like T cells, which might have a long-lasting impact on antitumor immunity (48–53). The sequential skin biopsies from patient 4349 demonstrated that “cold” tumor cells with no infiltrating T cells before cell infusion became heavily infiltrated with R175H-TCR^+^ T cells, suggesting TCR-engineered PBLs were able to home to the tumor site. Given the patient’s multiple prior chemotherapy regimens, it is unlikely that a single cycle of lymphodepleting chemotherapy contributed to the tumor regression.

This single case is insufficient to determine the causal relationship between the young and exhausted phenotypes of TCR-engineered PBL and TIL therapies, respectively, and their clinical responses. However, two patients (4141 and 4196; Table 2) who received autologous TIL therapies targeting p53^R175H^ (A*02:01-restricted) did not have clinical responses, whereas ACT with PBL transduced with the R175H-TCR, one of the three anti-p53^R175H^ TCRs isolated from patient 4196’s TIL, led to an objective response in patient 4349, indicating the importance of the phenotype of T cells for effective ACT. Furthermore, our preclinical testing of the R175H-TCR–transduced T cells showed that ACT using healthy donor PBLs led to complete regression of TYK-nu tumors in mice, whereas ACT using patient PBLs even at a higher dose level did not cause tumor regression, again pointing to the role of T-Cell phenotypes in successful ACT. Leukapheresis prior to systemic chemotherapies may help capture less differentiated/exhausted PBLs than collecting T cells that have undergone multiple cycles of depletion and reconstitution following cytotoxic chemotherapies (54).

Allogeneic use of TCRs targeting neoantigens and their potential toxicity have not previously been explored. Although a single case is insufficient to draw a firm conclusion, we did not detect any off-target toxicity of the transferred autologous PBLs expressing the allogeneic R175H-TCR except for CRS associated with on-target effects, similar to CAR T–cell therapies (55). Patient 4349’s tumor recurred with LOH of a portion of chromosome 6 spanning the HLA-A*02:01 locus. No HLA LOH was detected in the initial metastases we had resected to generate TILs; however, our data could not determine whether a relatively small subclonal population of tumor cells with HLA LOH existed before the ACT or whether the tumor cells rapidly evolved to lose HLA-A*02:01 following the ACT. Targeting multiple neoantigens with more than one HLA restriction and prescreening patients for intact HLA before ACT will likely be beneficial. Collectively, our data demonstrate the potential of ACT using the library of anti-mutant p53 TCRs to treat p53-mutated advanced cancers.

## Supplementary Material

SUPP FIGS 1 THRU 10

SUPP TABLES 1 THRU 7

## Figures and Tables

**Table 1.** Anti-mutant p53 TCR library.

TCR source (previous reporting)	*TP53* mutation	*TP53* mutation frequency (%)^a^	Tumor cell reactivity	HLA restriction	HLA frequency (%)^b^	Potentially treatable patient (%)
4141 (10,15); 4196 (12)	R175H	5.530	Yes	A*02:01	47.40	2.621
4273 (10,15)	R248W	3.218	ND	DPB1*02:01	27.30	0.878
4259	Y220C	1.790	Yes	A*02:01	47.40	0.848
4149 (10,11); 4343 (16)	Y220C	1.790	ND	DRB3*02:02	32.80	0.587
4285 (10,15)	R175H	5.530	ND	DRB1*13:01	10.00	0.553
4386	R273C	2.259	ND	DPB1*04:02	24.2^c^	0.547
4127 (10,11)	G245S	1.598	ND	DRB3*02:02	32.80	0.524
4259 (10)	Y220C	1.790	Yes^d^	DRB1*04:01	17.30	0.310
4266 (10,15)	R248W	3.218	Yes	A*68:01	6.38	0.205
4316	C135Y	0.426	ND	DRB1*07:01	26.84	0.114
4304	M237I	0.426	ND	DRB1*01:01	14.60	0.062
4316	C135Y	0.426	Yes	A*29:02	7.06	0.030
4324	T211I	0.032	Yes	C*06:02	18.64	0.006
4414	Y220D	0.011	ND	A*02:01	47.40	0.005
4114	C135R	0.043	ND	DRB1*11:01	10.9	0.005
4350	L111R	0.011	Yes	A*11:01	14	0.001
4356	Q331H	0.011	ND	B*40:01	11.00	0.001
4350	L111R	0.011	ND	DRB1*08:03	0.6	0.00006
4293	Y236S	0.011	ND	DRB3*02:01	0.33	0.00003
Sum						7.297

Abbreviation: ND, not determined.

a International Agency for Research on Cancer (IARC) TP53 Database (R20, July 2019, *N* = 9,386, colon, rectal, breast, pancreatic, esophageal, and ovarian cancers).

b Phenotype frequency of Caucasian populations in the United States; when the phenotype frequency is not available, twice the allele frequency is reported (http://www.allelefrequencies.net/).

c 60% to 80% in Hispanic populations, approximately 20% in African-American and Asian populations.

d Tumor cells transduced with DRA1/DRB1*04:01 were recognized while unmodified cells were not.

**Table 2.** Characteristics of 12 TIL and one TCR-engineered ACT.

Patient ID	Tumor type	*TP53* mutation	Treatment	HLA restriction	Number of IL2 doses	Number of pembrolizumab doses	% mutant p53-reactive cells in infusion product^a^	Total number of cells given to patients	Number of mutant p53-reactive cells given to patients	% persistence at 6 weeks post-ACT^a^	% PD1^+^ in infusion product	% TIM3^+^ in infusion product	% CD39^+^ in infusion product	% CD62L^+^ in infusion product	Response (months)
4114	Pancreatic	C135R	Autologous TIL	DRB1*11:01	2	0	18.9	2.14E+10	4.04E+09	Not detected	43.1	28.5	15.83	20.92	NR
4127	Ovarian	G245S	Autologous TIL	DRB3*02:02	5	2	2.8	1.43E+11	4.00E+09	Not detected	16.66	33.83	71.8	15.94	PR (4)
4141	Colon	R175H	Autologous TIL	A*02:01	4	4	2.2	6.90E+10	1.52E+09	0.05	46.2	32	87.4	29.24	NR
4149	Ovarian	Y220C	Autologous TIL	DRB3*02:02	5	4	11.1	3.71E+10	4.12E+09	Not detected	58.7	15.27	93.2	6.8	NR
4196 (12)	Colon	R175H	Autologous TIL	A*02:01	0	4	3.3	9.18E+10	3.03E+09	0.02	NA	NA	NA	NA	NR
4266	Colon	R248W	Autologous TIL	A*68:01:02	6	0	50.8	1.01E+11	5.13E+10	0.19	64	89.5	98.8	0.89	NR
4273	Rectal	R248W	Autologous TIL	DPB1*02:01:02	2	2^c^	6.75	1.17E+11	7.90E+09	NA	28.4	42.7	96.3	4.03	NR
4285	Colon	R175H	Autologous TIL	DRB1*13:01:01	6	0	2.43	6.96E+10	1.69E+09	Not detected	55.9	56.5	76.11	2.08	NR
4304	Colorectal	M237I	Autologous TIL	DRB1*01:01	4	4	13 (3 TCRs)	8.97E+10	1.17E+10	0.01	30.6	19.32	97.4	2.79	NR
4324	Colorectal	T211I	Autologous TIL	C*06:02	3	4	45 (2 TCRs)	8.49E+10	3.82E+10	1.45	29.8	75.7	29.8	5.02	NR
4343 (16)	Breast	Y220C	Autologous TIL	DRB3*02:02	4	4	1	7.68E+10	7.68E+08	0.002	63.2	NA	97.1	9.73	PR (6)
4350	Colon	L111R	Autologous TIL	A*11:01	5	2	11	6.69E+10	7.36E+09	0.115	30.9	3.37	94.2	3.37	NR
4349	Breast	R175H	TCR-engineered PBL	A*02:01	0	1^c^	64^b^	5.30E+10	3.39E+10	14.4^b^	13	21.7	52	51.5	PR (6)

Abbreviation: NA, not available; NR, no response.

a Mutant p53–reactive T-cell frequency determined by TCRB sequencing except for 4349 (Adaptive Biotechnology).

b Frequency determined by flow cytometry against the murine TCRβ constant region.

c These patients received pembrolizumab post-ACT.
