# Supplementary material for: Adoptive Cellular Therapy with Autologous Tumor-Infiltrating Lymphocytes and T-cell Receptor-Engineered T Cells Targeting Common p53 Neoantigens in Human Solid Tumors
Source: Cancer Immunol Res. Author manuscript; Available in PMC 2022 Aug 7. (PMC9357191; doi:10.1158/2326-6066.CIR-22-0040)
Supplement: SUPP FIGS 1 THRU 10 [file NIHMS1822393-supplement-SUPP_FIGS_1_THRU_10.pdf]

Supplementary Figures for Kim et al.

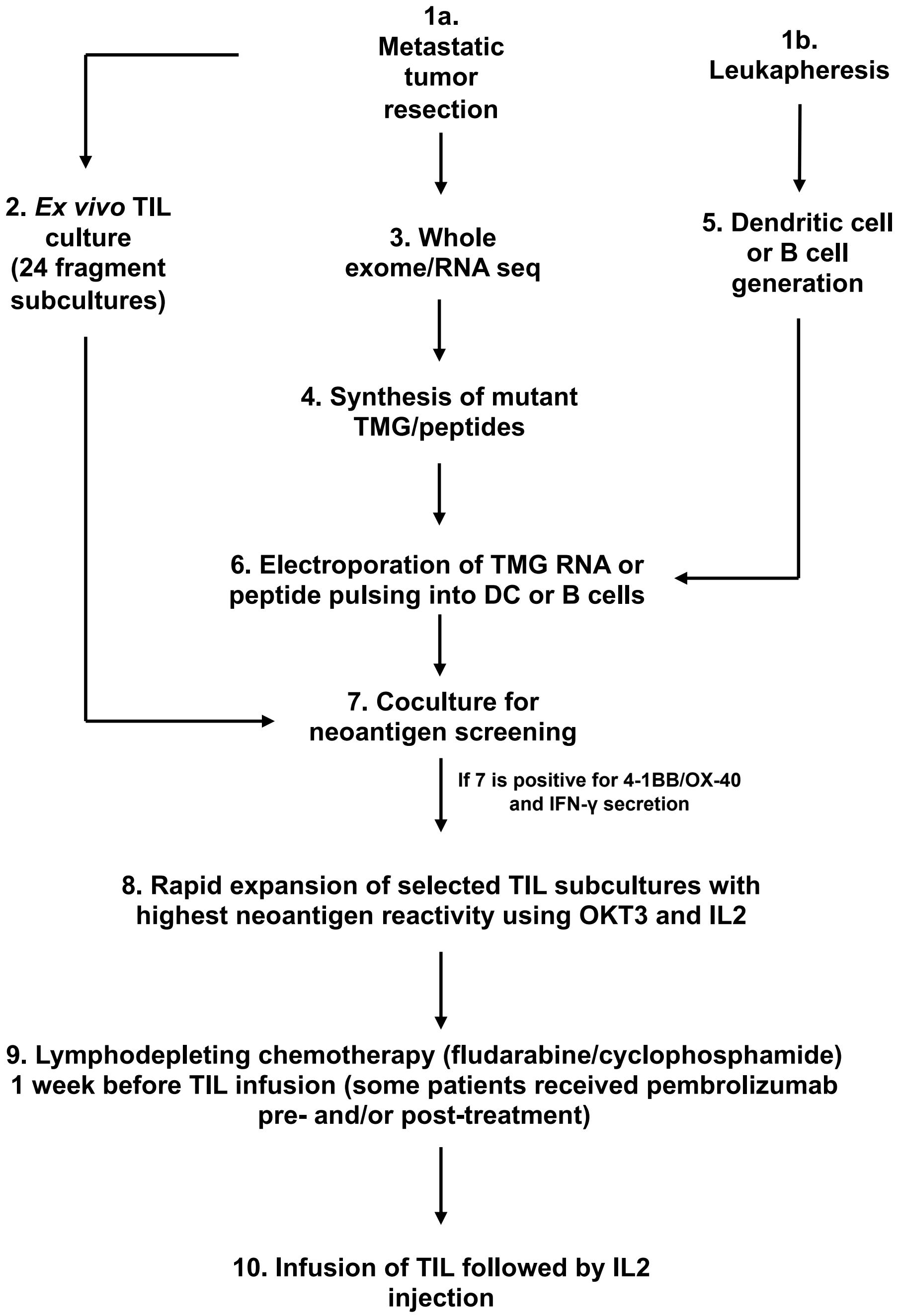

Supplementary Figure S1. Processes for development and treatment with autologous TIL infusion products targeting p53 neoantigens. Diagram showing the process for TIL infusion product development and patient treatment. See also Fig. 1A.

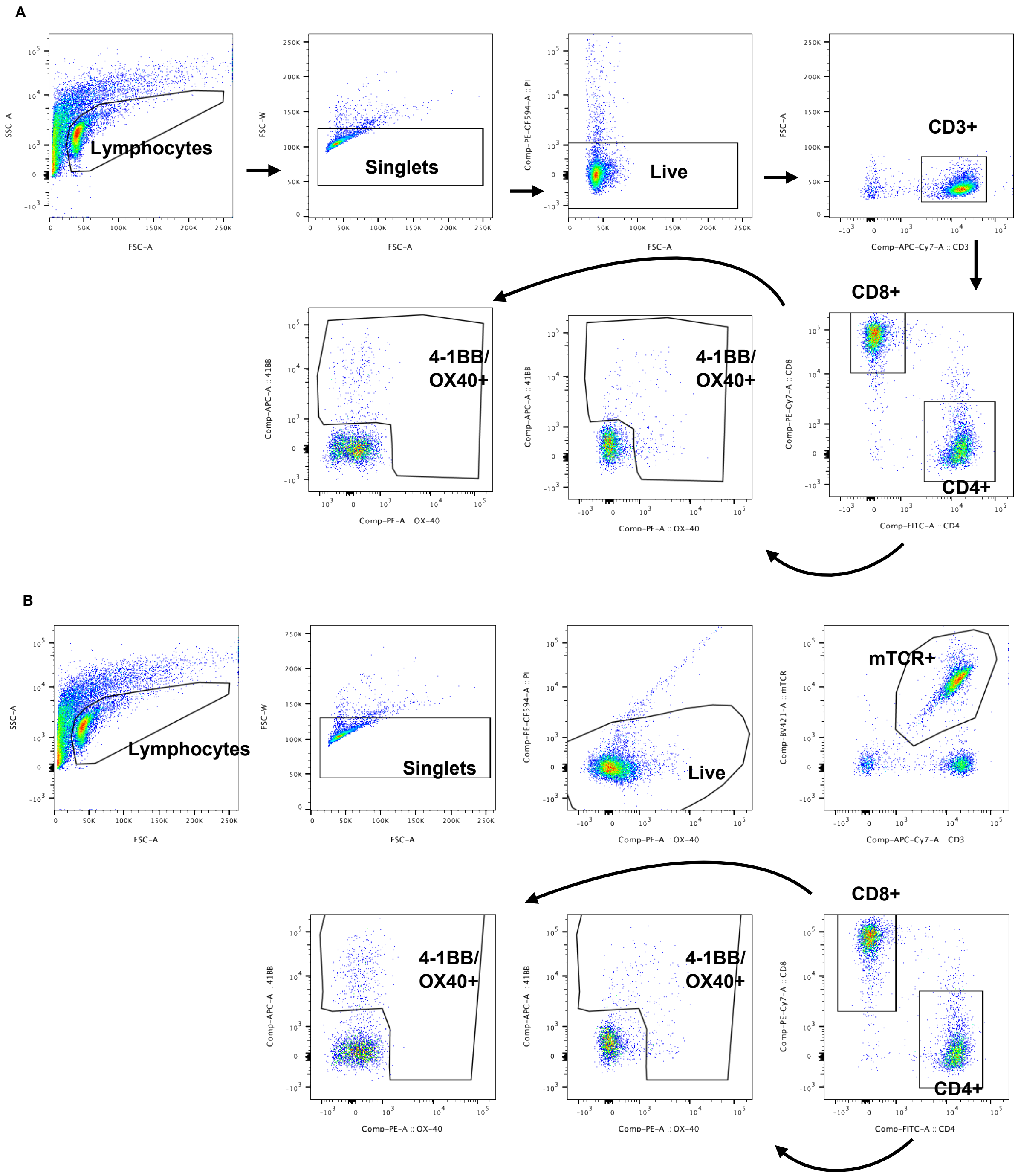

Supplementary Figure S2. Gating strategy for flow cytometric analysis of TIL (A) and PBLs engineered with a TCR (B).

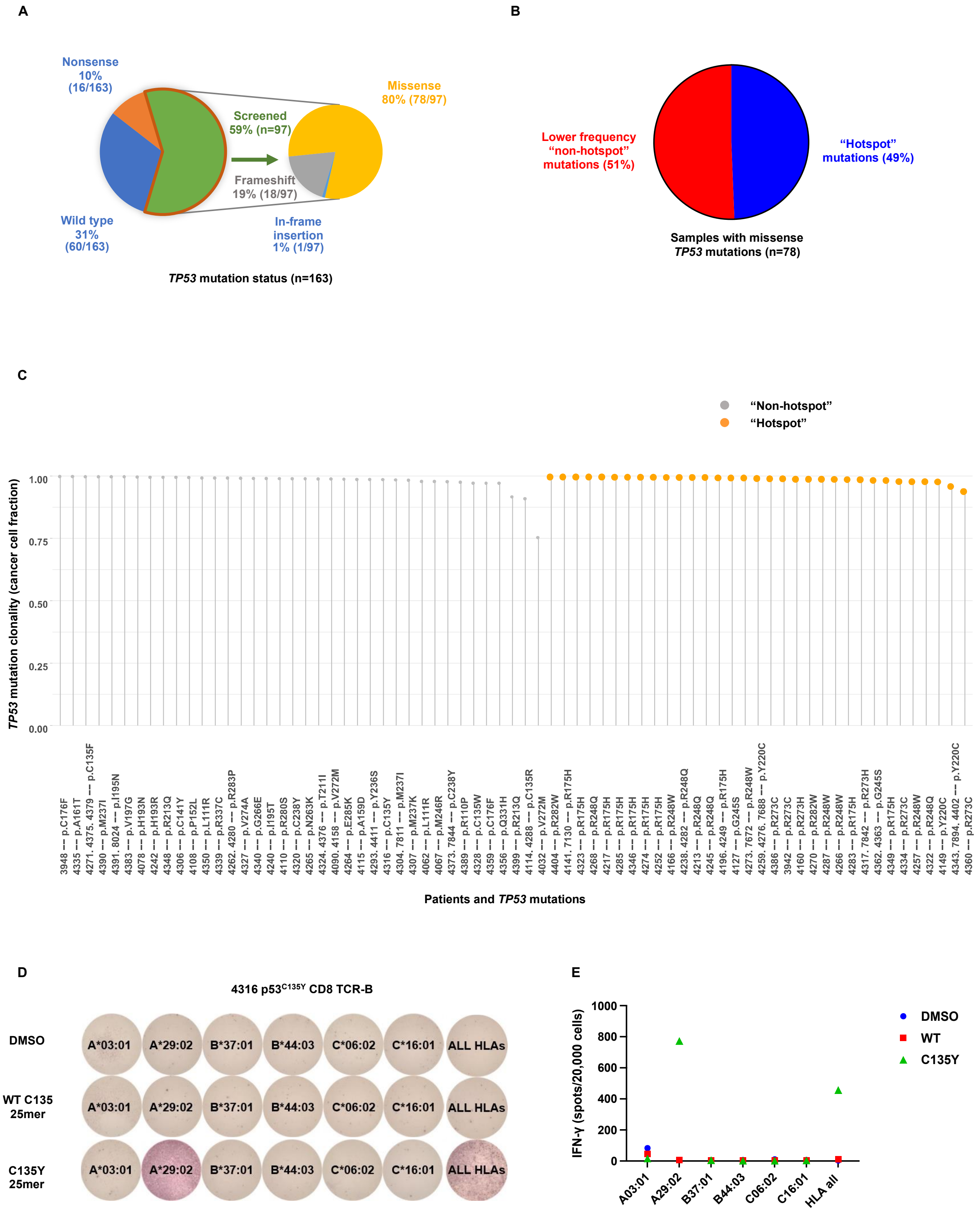
A
Nonsense 10% (16/163)
Wild type 31% (60/163)
Screened 59% (n=97)
Missense 80% (78/97)
Frameshift 19% (18/97)
In-frame insertion 1% (1/97)
TP53 mutation status (n=163)
B
Lower frequency “non-hotspot” mutations (51%)
“Hotspot” mutations (49%)
Samples with missense TP53 mutations (n=78)
C
“Non-hotspot”
“Hotspot”
TP53 mutation clonality (cancer cell fraction)
1.00
0.75
0.50
0.25
0.00
3948 --- p.C176F
4335 --- p.A161T
4271. 4375. 4379 --- p.C135F
4390 --- p.M237I
4391. 8024 --- p.I195N
4383 --- p.V197G
4078 --- p.H193N
4242 --- p.H193R
4348 --- p.R213Q
4306 --- p.C141Y
4108 --- p.P152L
4350 --- p.L111R
4339 --- p.R337C
4262. 4280 --- p.R283P
4327 --- p.V274A
4340 --- p.G266E
4240 --- p.I195T
4110 --- p.R280S
4320 --- p.C238Y
4265 --- p.N263K
4324. 4376 --- p.T211I
4090. 4158 --- p.V272M
4264 --- p.E285K
4115 --- p.A159D
4293. 4411 --- p.Y236S
4316 --- p.C135Y
4304. 7811 --- p.M237I
4307 --- p.M237K
4062 --- p.L111R
4067 --- p.M246R
4373. 7844 --- p.C238Y
4389 --- p.R110P
4328 --- p.C135W
4359 --- p.C176F
4356 --- p.Q331H
4399 --- p.R213Q
4114. 4288 --- p.C135R
4032 --- p.V272M
4404 --- p.R282W
4141. 7130 --- p.R175H
4323 --- p.R175H
4268 --- p.R248Q
4217 --- p.R175H
4285 --- p.R175H
4346 --- p.R175H
4274 --- p.R175H
4252 --- p.R175H
4166 --- p.R248W
4238. 4282 --- p.R248Q
4213 --- p.R248Q
4245 --- p.R248Q
4196. 4249 --- p.R175H
4127 --- p.G245S
4273. 7672 --- p.R248W
4259. 4276. 7688 --- p.Y220C
4386 --- p.R273C
3942 --- p.R273C
4160 --- p.R273H
4270 --- p.R282W
4287 --- p.R248W
4266 --- p.R248W
4283 --- p.R175H
4317. 7842 --- p.R273H
4362. 4363 --- p.G245S
4349 --- p.R175H
4334 --- p.R273C
4257 --- p.R248W
4322 --- p.R248Q
4149 --- p.Y220C
4343. 7894. 4402 --- p.Y220C
4360 --- p.R273C
Patients and TP53 mutations
D
4316 p53C135Y CD8 TCR-B
DMSO
WT C135 25mer
C135Y 25mer
A*03:01
A*29:02
B*37:01
B*44:03
C*06:02
C*16:01
ALL HLAs
E
IFN-γ (spots/20,000 cells)
1000
800
600
400
200
0
A03:01
A29:02
B37:01
B44:03
C06:02
C16:01
HLA all
DMSO
WT
C135Y

Supplementary Figure S3. Characteristics of *TP53* mutations in the Surgery Branch cohort and HLA restriction of CD8 TCR-B isolated from patient 4316.

A, Pie chart showing the frequencies of somatic *TP53* mutations within the Surgery Branch patient cohort (n=163). Patients with missense, frameshift and in-frame insertion mutations in *TP53* that consisted 59% of the patients were subjected to the immunogenicity screening for *TP53* mutations. **B,** Pie chart showing the frequencies of "hotspot" and "non-hotspot" *TP53* missense mutations within the Surgery Branch cohort (n=78). **C,** Clonality of "hotspot" and "non-hotspot" *TP53* mutations. The cancer cell fraction values for *TP53* mutations generated from the whole exome sequencing results are plotted. Each dot represents the average clonality for three or more tumor fragments from a single patient. **D**, COS7 cells individually transfected with patient 4316's own HLA class I were pulsed with DMSO, wild-type or p53C135Y 25mer peptides and co-cultured with CD8 TCR-B-expressing healthy donor PBLs. ELISpot measurement of IFN-γ secretion is shown (N=1). **E**, IFN-γ spots in **D** are quantified.

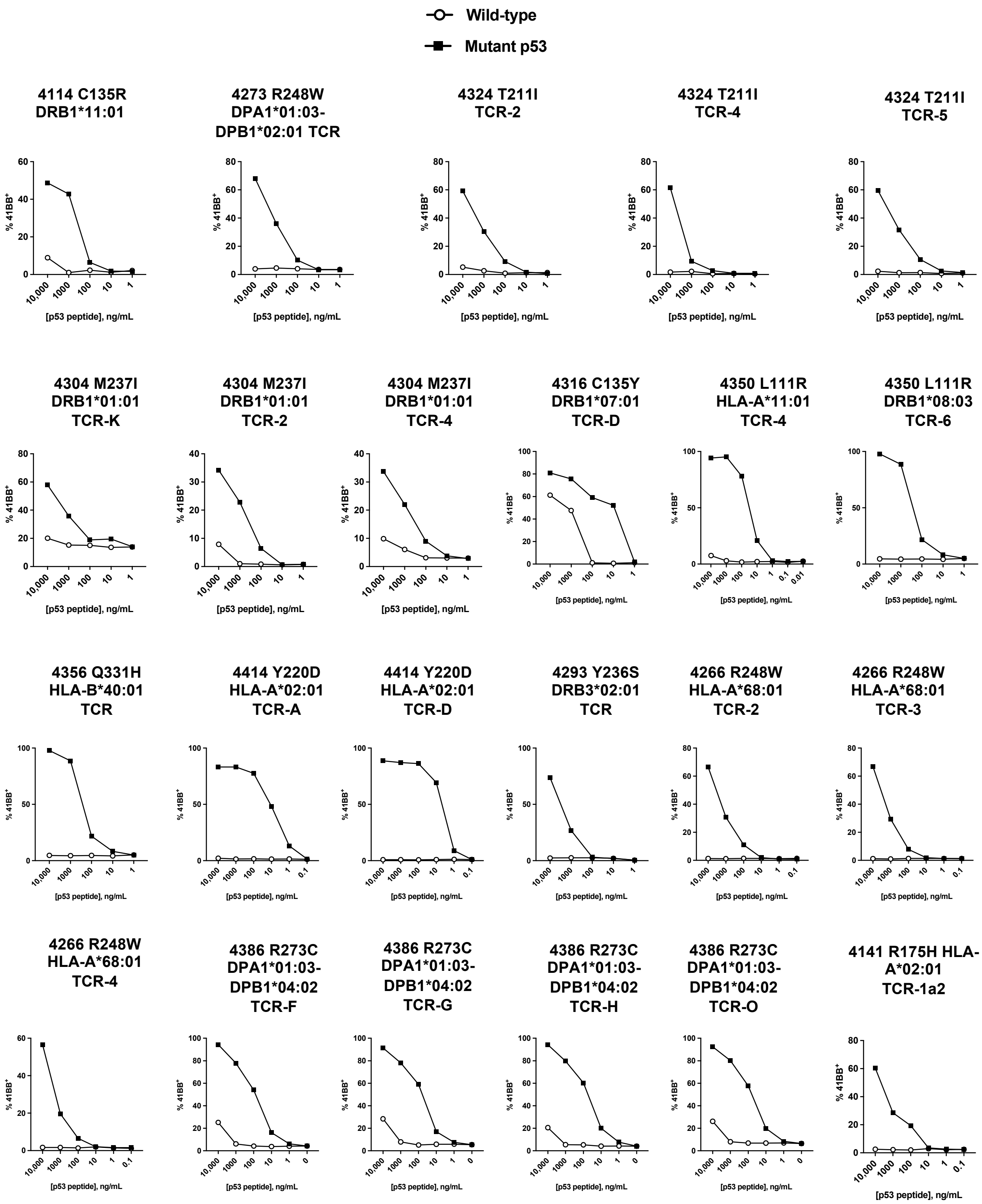

Wild-type
Mutant p53
4114 C135R DRB1*11:01
4273 R248W DPA1*01:03-DPB1*02:01 TCR
4324 T211I TCR-2
4324 T211I TCR-4
4324 T211I TCR-5
4304 M237I DRB1*01:01 TCR-K
4304 M237I DRB1*01:01 TCR-2
4304 M237I DRB1*01:01 TCR-4
4316 C135Y DRB1*07:01 TCR-D
4350 L111R HLA-A*11:01 TCR-4
4350 L111R DRB1*08:03 TCR-6
4356 Q331H HLA-B*40:01 TCR
4414 Y220D HLA-A*02:01 TCR-A
4414 Y220D HLA-A*02:01 TCR-D
4293 Y236S DRB3*02:01 TCR
4266 R248W HLA-A*68:01 TCR-2
4266 R248W HLA-A*68:01 TCR-3
4266 R248W HLA-A*68:01 TCR-4
4386 R273C DPA1*01:03-DPB1*04:02 TCR-F
4386 R273C DPA1*01:03-DPB1*04:02 TCR-G
4386 R273C DPA1*01:03-DPB1*04:02 TCR-H
4386 R273C DPA1*01:03-DPB1*04:02 TCR-O
4141 R175H HLA-A*02:01 TCR-1a2
% 41BB+
[p53 peptide], ng/mL

Supplementary Figure S4. Avidity of the library of the anti-mutant p53 TCRs targeting p53 neoantigens. *TCRA/B* sequences were reconstructed into an MSGV1 retroviral backbone and retrovirus was generated for the individual TCRs from Table 1. Healthy donor PBLs were transduced to express the TCRs. Exogenous TCR expression was validated by positive staining of an H57-597 antibody against the mouse *TCRB* constant region (mTCR). Finally, TCR-engineered PBLs were titrated against imDC or B cells pulsed with a serially diluted wild-type or mutant peptide (either 25mer or minimal epitopes of 9 to 12mer). The frequencies of 4-1BB$^+$ cells of mTCR$^+$/CD4$^+$ or mTCR$^+$/CD8$^+$ cells are shown. Data for the TCRs that were previously reported are not shown. N=1, the experiments in A-E were independently repeated at least once.

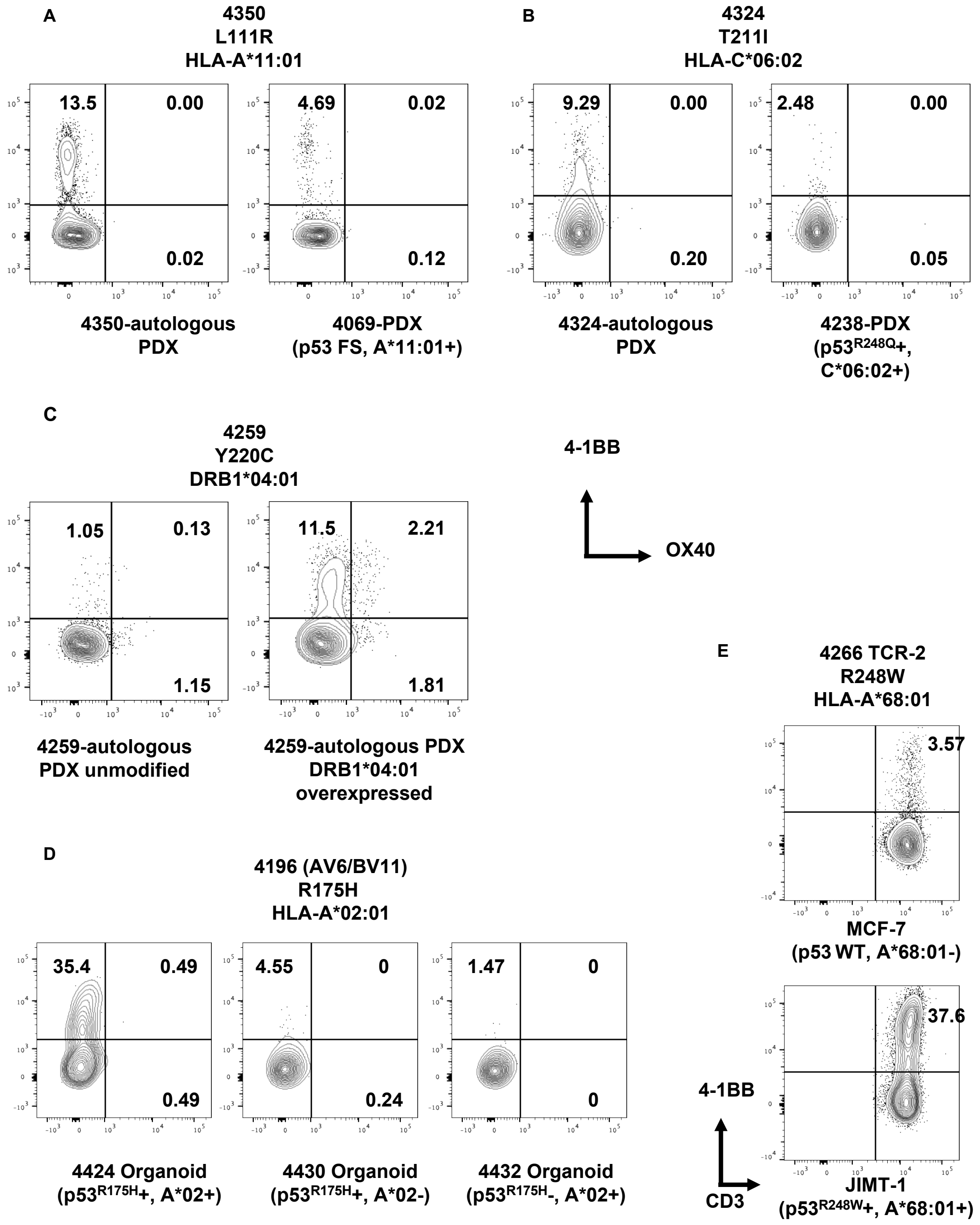

Supplementary Figure S5. Recognition of tumor cells by neoantigen-reactive T cells.

A-E, Autologous or healthy donor T cells expressing above listed TCRs were cocultured with autologous PDX cells or allogeneic cells with matching *TP53* mutations and HLA for 18 to 24 hours. Irrelevant PDX cells or commercially available cell lines without corresponding *TP53*

mutations or HLA were included as negative controls. The flow cytometry data are pre-gated for CD8+ (**A**, **B, D** and **E**) or CD4 (**C**), and 4-1BB and OX40 expression is shown (**E** is plotted for 4-1BB and CD3). **C**, Unmodified 4259 autologous PDX cells or 4259 PDX cells transduced with DRA1/DRB1*04:01 were used. FS, frame-shift mutations. N=1, the experiments in A-E were independently repeated at least once.

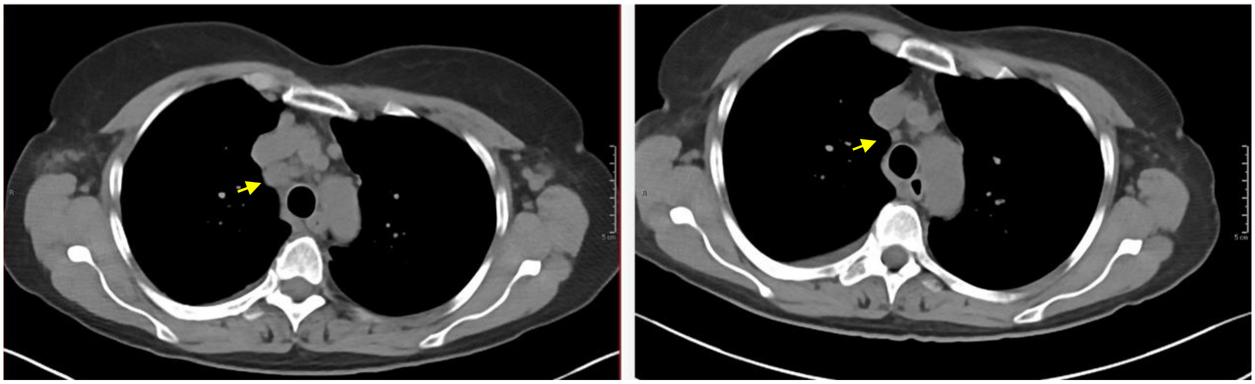

Supplementary Figure S6. Computed tomography of chest of patients 4127.

Contrast-enhanced computed tomographic scans of the chest of patient 4127 before (left) and approximately 3 months after the infusion of the autologous TIL (right). Arrows highlight the right paratracheal lymph node before and after therapy.

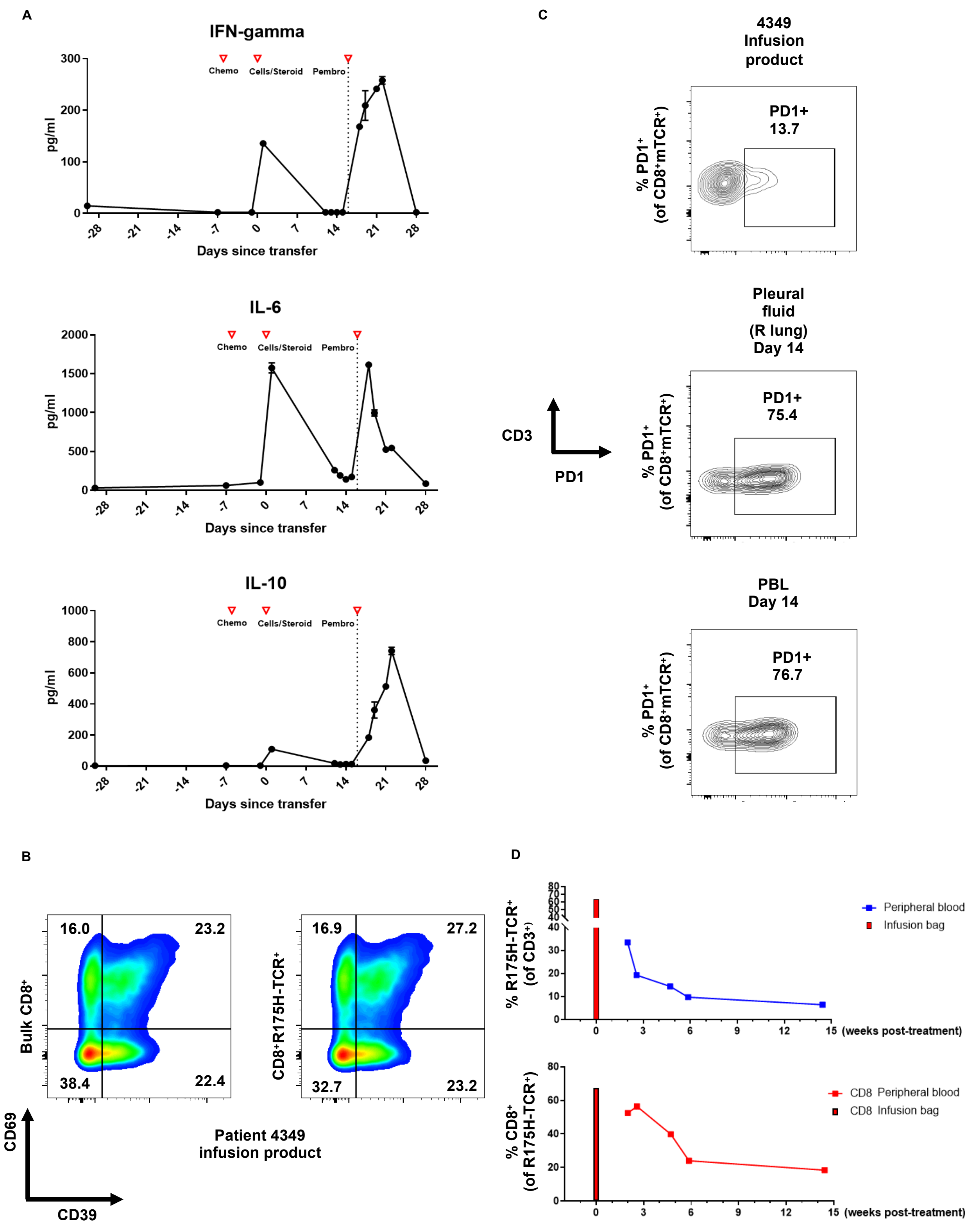
A
IFN-gamma
Chemo
Cells/Steroid
Pembro
pg/ml
Days since transfer
IL-6
IL-10
B
16.0
23.2
38.4
22.4
Bulk CD8⁺
16.9
27.2
32.7
23.2
CD8⁺R175H-TCR⁺
CD69
CD39
Patient 4349
infusion product
C
4349
Infusion
product
PD1+
13.7
% PD1⁺
(of CD8⁺mTCR⁺)
Pleural
fluid
(R lung)
Day 14
PD1+
75.4
CD3
PD1
PBL
Day 14
PD1+
76.7
D
% R175H-TCR⁺
(of CD3⁺)
Peripheral blood
Infusion bag
(weeks post-treatment)
% CD8⁺
(of R175H-TCR⁺)
CD8 Peripheral blood
CD8 Infusion bag

Supplementary Fig. S7. Analysis of T cells and cytokines for patient 4349. A, Phenotypic analysis of antigen-specific (R175H-TCR$^+$) or bulk CD8+ T cells from the infusion products for patient 4349 by flow cytometry. Bulk CD8$^+$ T cells (left) or CD8$^+$R175H-TCR$^+$ cells (right) were stained for CD39 and CD69. **B**, Serum cytokine levels of IFN-γ (top), IL-6 (middle), and IL-10 (bottom) were measured by a flow cytometry-based LEGENDplex assay. **C,** Flow cytometric analysis of patient 4349's infusion product (top), pleural fluid at day 14 (middle), and PBL at day 14 (bottom) for PD1 expression. **D,** Persistence of R175H-TCR-expressing T cells by flow cytometric detection of R175H-TCR$^+$ T cells (upper panel) and CD8$^+$ T cells of R175H-TCR$^+$ T cells (bottom panel).

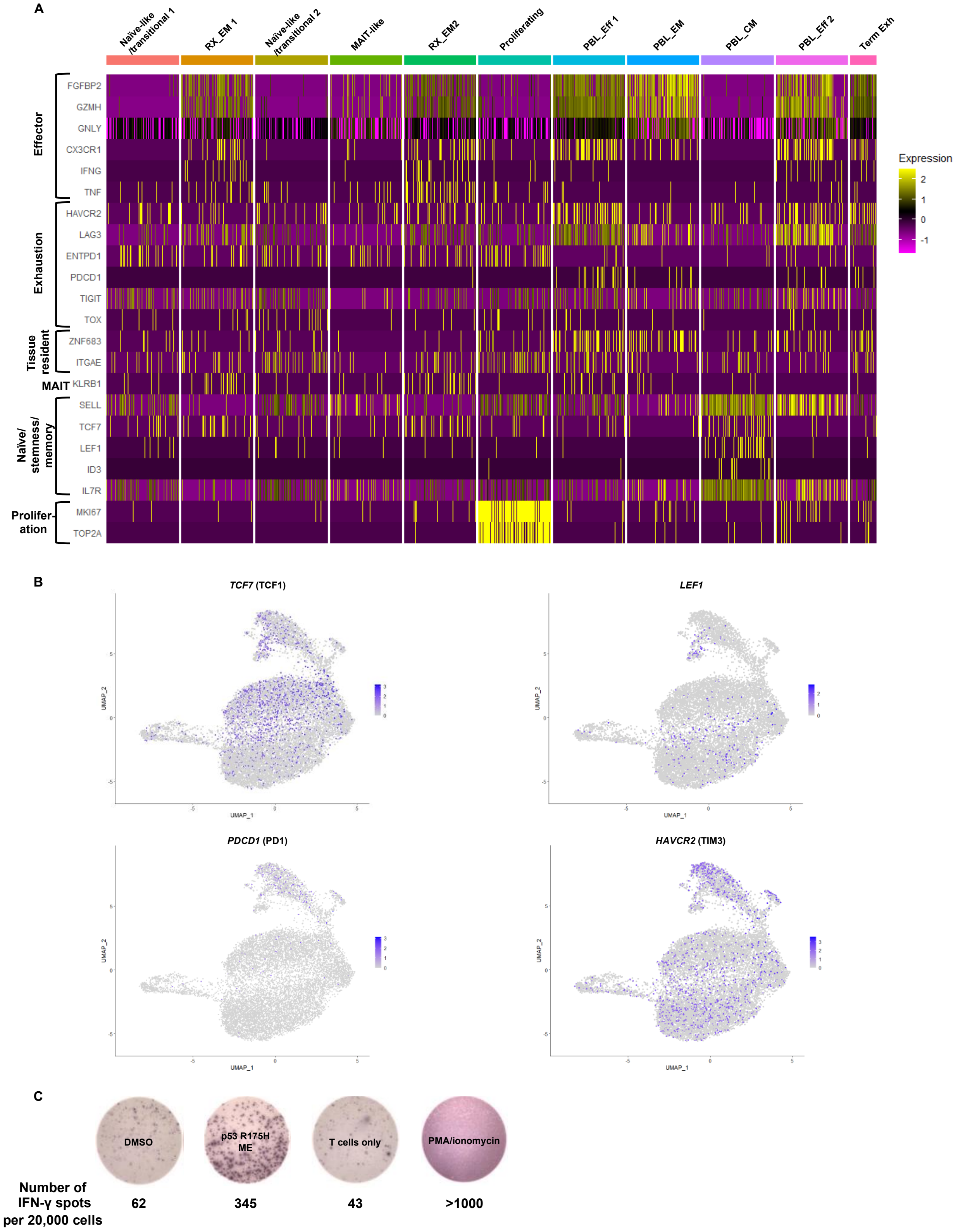
A
Naïve-like /transitional 1
RX_EM 1
Naïve-like /transitional 2
MAIT-like
RX_EM2
Proliferating
PBL_Eff 1
PBL_EM
PBL_CM
PBL_Eff 2
Term Exh
Effector
FGFBP2
GZMH
GNLY
CX3CR1
IFNG
TNF
Exhaustion
HAVCR2
LAG3
ENTPD1
PDCD1
TIGIT
TOX
Tissue resident
ZNF683
ITGAE
MAIT
KLRB1
Naive/ stemness/ memory
SELL
TCF7
LEF1
ID3
IL7R
Prolifer-ation
MKI67
TOP2A
Expression
2
1
0
-1
B
TCF7 (TCF1)
LEF1
PDCD1 (PD1)
HAVCR2 (TIM3)
UMAP_1
UMAP_2
C
DMSO
p53 R175H ME
T cells only
PMA/ionomycin
Number of IFN-γ spots per 20,000 cells
62
345
43
>1000

Supplementary Fig. S8. Analysis of patient 4349's infusion product (RX) and PBL samples post-ACT. A, Heatmap of selected genes across the clusters in Fig. 4F. Expression from each single cell was down sampled to 100 cells per cluster for visibility. **B,** Expression of indicated genes overlaid on the UMAP projection of RX cells and PBL_6w cells. **C,** Functional analysis of PBL sample at 4 months post-ACT. Peripheral blood mononuclear cells were isolated by Ficoll and were subject to co-culture with autologous imDC pulsed with the $p53^{R175H}$ ME or DMSO. T cells only and PMA/ionomycin conditions were included as negative and positive controls, respectively.

A

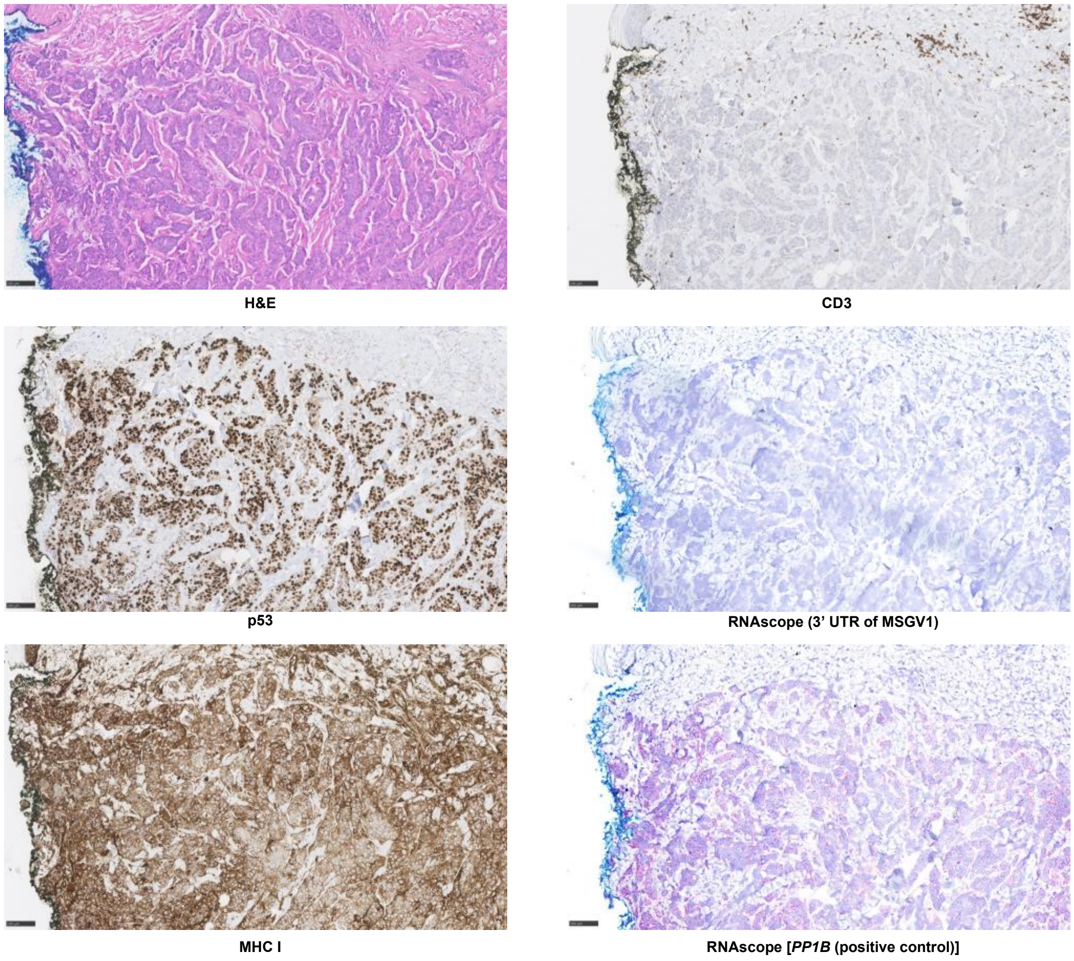
H&E
CD3
p53
RNAscope (3' UTR of MSGV1)
MHC I
RNAscope [PP1B (positive control)]

B

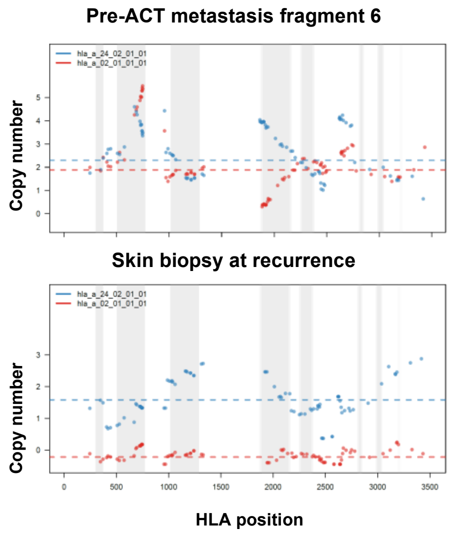
Pre-ACT metastasis fragment 6
hla_a_24_02_01_01
hla_a_02_01_01_01
Copy number
Skin biopsy at recurrence
Copy number
HLA position

C

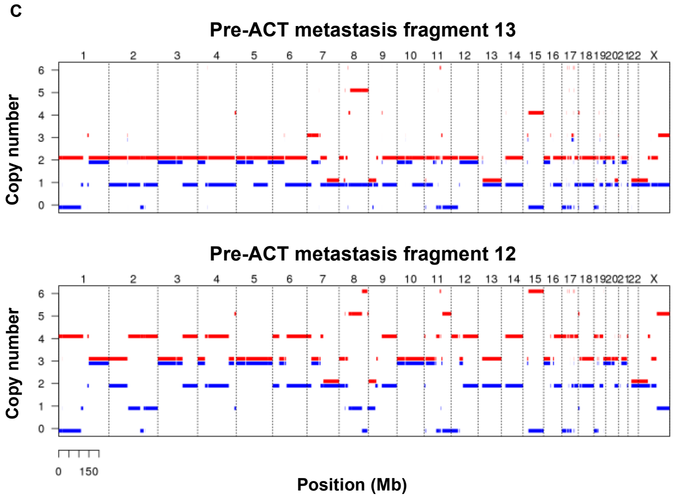
Pre-ACT metastasis fragment 13
Copy number
Pre-ACT metastasis fragment 12
Copy number
Position (Mb)

Supplementary Fig. S9. Immunohistochemical and genomic analysis of the skin biopsies of patient 4349's progressing lesion and the pre-ACT metastases. A, Skin biopsies from patient 4349 at day 209 post-ACT were analyzed by an immunohistochemical and RNAscope analysis. RNAscope against *PP1B* was included as a positive control to ensure the quality of RNA of the specimen. Scale bar, 100 μm. **B,** Copy number analysis at the chromosome level. **C,** Haplotype specific copy number analysis for the HLA-A locus. Exons are shaded in gray and each dot represents a copy number for a single nucleotide polymorphism corrected for Log R ratio, B allele frequency, tumor purity and tumor ploidy. Dotted lines represent the median allele copy number.

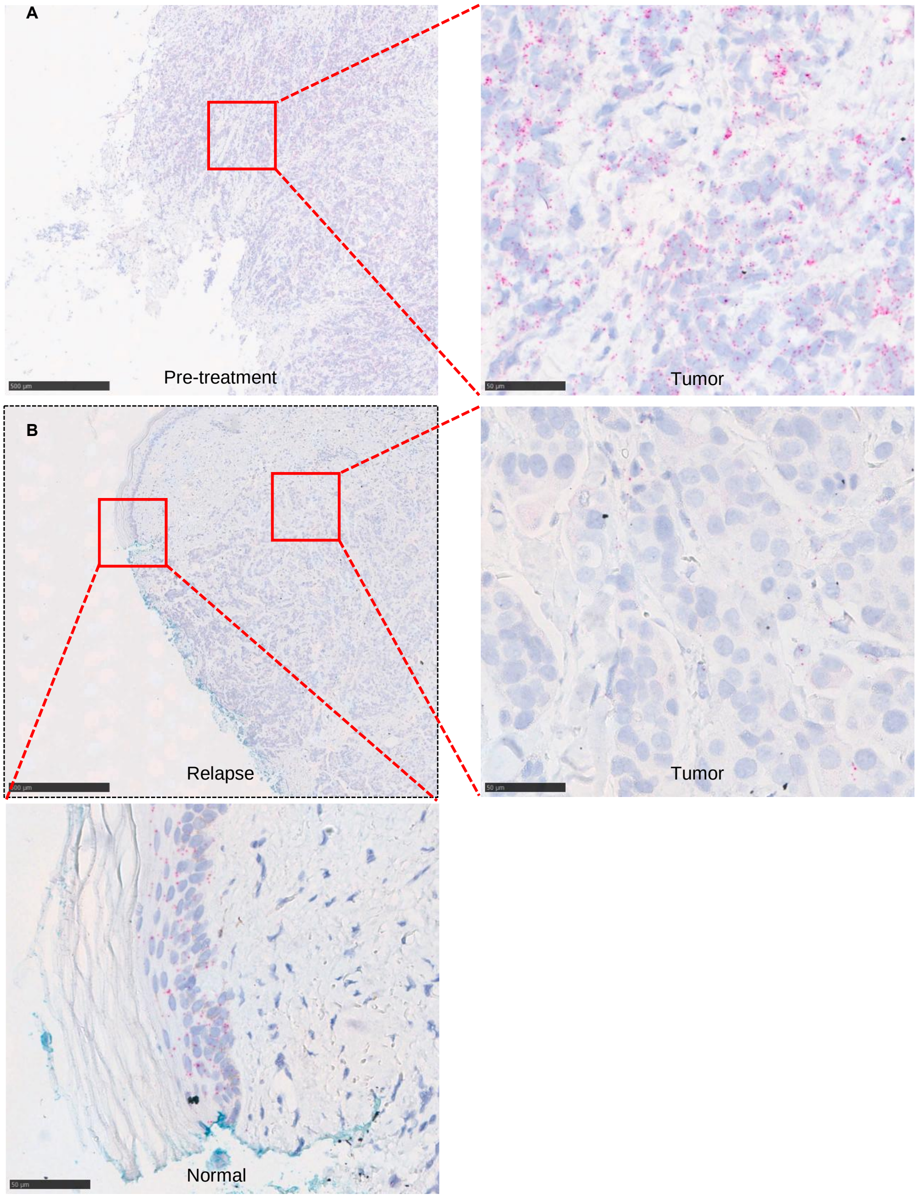

Supplementary Fig. S10. Detection of HLA-A*02:01 by RNAscope in the pre-ACT and progressing lesion biopsies from patient 4349. Skin biopsies from patient 4349 before the ACT (**A**) and at recurrence at day 209 post-ACT (**B**) were analyzed by an RNAscope analysis using a probe against HLA-A*02:01. **B,** From the relapse biopsy, the normal epidermis with intact HLA-A*02:01 expression (bottom panel) and the tumor cells with a lack of HLA-A*02:01 expression (right panel) are shown. Scale bar, left panel in A and top left panel in B: 500 µm; right panel in A and top right and bottom panel in B: 50 µm.
